# Effect of In-Person Delivered Behavioural Interventions in People with Multimorbidity: Systematic Review and Meta-analysis

**DOI:** 10.1007/s12529-022-10092-8

**Published:** 2022-04-28

**Authors:** Alessio Bricca, Madalina Jäger, Marie Johnston, Graziella Zangger, Lasse K. Harris, Julie Midtgaard, Søren T. Skou

**Affiliations:** 1 Research Unit for Musculoskeletal Function and Physiotherapy, Department of Sports Science and Clinical Biomechanics, University of Southern Denmark, 5230 Odense M, Denmark; 2 The Research Unit PROgrez, Department of Physiotherapy and Occupational Therapy, Næstved-Slagelse-Ringsted Hospitals, Region Zealand, 4200 Slagelse, Denmark; 3 Health Psychology Group, Institute of Applied Health Sciences, University of Aberdeen, Aberdeen, Scotland; 4 University Hospitals Centre for Health Care Research (UCSF), Copenhagen University Hospital Rigshospitalet, Copenhagen, Denmark; 5 Department of Public Health, Faculty of Health and Medical Sciences, University of Copenhagen, Copenhagen, Denmark

**Keywords:** Physical activity, Behavioural therapy, Multimorbidity, Function, Disability, Health

## Abstract

**Background:**

To investigate the effect of in-person delivered behavioural interventions in people with multimorbidity and which behaviour change techniques (BCTs), targeting lifestyle behaviours, are associated with better outcomes.

**Methods:**

Systematic review of randomised controlled trials. We searched MEDLINE, EMBASE, CENTRAL, and CINAHL and screened reference list of reviews including people with multimorbidity, registries, and citation tracking of included studies. Meta-analyses using random-effects model to assess the effect of behavioural interventions and meta-regression analyses and effectiveness ratios to investigate the impact of mediators on effect estimates. Cochrane ‘Risk of Bias Tool’ 2.0 and the GRADE assessment to evaluate the overall quality of evidence.

**Results:**

Fourteen studies involving 1,378 people. Behavioural interventions had little to no effect on physical activity (standardised mean difference 0.38, 95% CI −0.12–0.87) and the effect on weight loss was uncertain (BMI mean difference −0.17, 95% CI −1.1–0.83) at the end-treatment follow-up. Small improvements were seen in health-related quality of life (SMD 0.29, 95% CI 0.17–0.42) and physical function (SMD 0.42, 95% CI 0.12–0.73), and moderate improvements were seen for depression symptoms (SMD −0.70, 95% CI −0.97–0.42). Studies using the BCTs ‘action planning’ and ‘social support (practical)’ reported greater physical activity and weight loss.

**Conclusions:**

Behavioural interventions targeting lifestyle behaviours may improve health-related quality of life and physical function, and reduce depression, whereas little to no effect was achieved on physical activity and weight loss in people with multimorbidity. However, the evidence for physical activity and weight loss were of low quality and the end-treatment benefits diminished over time.

**Supplementary Information:**

The online version contains supplementary material available at 10.1007/s12529-022-10092-8.

## Introduction

Living with multiple chronic conditions (i.e., multimorbidity) is very common not only in the elderly population [1]. Compared to people living with single chronic conditions, people with multimorbidity are at increased risk of dying prematurely, being admitted to and have an increased length of stay in the hospital [2, 3], have poorer physical and psychosocial health, higher intake of multiple drugs, and increased health care utilisation [4, 5]. This challenges the current usual care of people with multimorbidity focusing on single-disease management approaches as opposed to individualised, multimorbidity care [6, 7].

Individualised care for people with multimorbidity includes recommendations related to a healthy lifestyle [8]. Physical activity is low in people with multimorbidity [9], although being a key behaviour for survival and overall health alongside a healthy diet, not smoking, and low alcohol consumption [10]. While interventions targeting lifestyle behaviours, including physical activity and diet, benefit people with single chronic conditions [11] and those at risk of developing chronic conditions [12], less is known about their effects in people with multimorbidity, which are often excluded from clinical trials [13]. Some behaviour change techniques (BCTs) that is ‘an observable, replicable and irreducible component of an intervention designed to alter or redirect causal processes that regulate behaviour such as action planning, self-monitoring and goal setting’ [14] are strongly associated with improved health behaviours in people without chronic conditions [11]. The self-regulatory process may be the driver of these benefits; however, the association between BCTs and health behaviours in people with multimorbidity is unclear, including why some BCTs may be more effective than others.

Due to the complexity of multimorbidity, to provide individualised care, it has been suggested to focus on specific combinations of conditions, linked by specific risk factors (e.g., inactivity) and pathogenesis (e.g., systemic low grade inflammation) [15–18]. Osteoarthritis of the knee or hip, hypertension, type 2 diabetes, depression, heart failure, ischemic heart disease, and chronic obstructive pulmonary disease are among the leading causes of global disability [19]. Given these conditions are triggered by physical inactivity and systemic low grade inflammation, interventions targeting physical activity have the potential to improve the physical and psychosocial health of this population, thanks to the anti-inflammatory effect of physical activity [20]. However, to our knowledge, no systematic reviews have investigated the effect of behavioural interventions and BCTs in the aforementioned combinations of (medical) conditions. While the BCTs that are effective for people without chronic conditions may well work also for people with multimorbidity, it is important to gather direct evidence (i.e., evidence delivered to the populations in which we are interested) to generalise the result to the multimorbidity population. Providing a summary of the effect of behavioural interventions in this population and identifying effective BCTs to improve lifestyle behaviours and the physical and psychosocial health of people with multimorbidity may also help to individualise treatment options for this population.

This systematic review aims to investigate the effect of behavioural interventions and BCTs on behavioural, physical, and psychosocial outcomes in people with at least two of the following chronic conditions: osteoarthritis of the knee or hip, hypertension, type 2 diabetes, depression, heart failure, ischemic heart disease, and chronic obstructive pulmonary disease.

## Methods

We followed the Cochrane Handbook recommendations for performing systematic reviews [21] and and the Methodological Expectations of Cochrane Intervention Reviews (MECIR) for performing this systematic review [22]. This systematic review was reported following the Preferred Reporting Items for Systematic Reviews and Meta-analyses (PRISMA) guidelines [23]. The protocol for this systematic review was made publicly available on the Open Science Framework website [24] before the title and abstract screening phase was initiated.

### Eligibility Criteria

#### Population

The review included RCTs published in peer-reviewed journals including adults (≥ 18 years old), and including people diagnosed with at least two of the following conditions (based on clinical records or screening with validated instruments): osteoarthritis of the knee or hip, heart failure, ischemic heart disease, hypertension (systolic blood pressure ≥ 140 and diastolic blood pressure ≥ 90), type 2 diabetes mellitus, chronic obstructive pulmonary disease, and depression as defined by the studies or calculated from baseline participant characteristics. As an example, we only included studies in people with depressive symptoms which required treatment. This is in line with clinical guidelines for depression, highlighting that a patient with any degree of depression severity is considered to have depression if offered a treatment [25]. This approach prevented us from including studies that included people that did not have clinical depression.

#### Interventions

Interventions were included if they targeted self-directed health behaviours. For example, multifaceted interventions to increase physical activity and/or weight loss, among other lifestyle behaviours, delivered by health care providers in a group or one-to-one format.

### BCT Coding

Interventions were coded for BCTs using the Behaviour Change Technique Taxonomy (v1) [14] by two researchers (MJä and GZ). The BCT taxonomy is a reliable method for specifying, interpreting, and implementing the active ingredients of interventions to change behaviours. The BCT Taxonomy v1 contains a cross-domain, hierarchically structured taxonomy of 93 distinct BCTs with labels, definitions, and examples [14], and it is a useful method for both research and practice. Each of the researchers coded all the interventions independently. Disagreements were resolved through discussion, and a third reviewer (MJo) mediated where a consensus could not be reached. MJä and GZ are trained in using the taxonomy and practised coding BCTs before this task via the online BCT community (https://www.bct-taxonomy.com/). All the intervention elements that contain specific BCT were coded. Only interventions (components) that closely correspond to the definitions of the BCTs provided in the taxonomy were coded. Authors were contacted if data was missing or unclear, and intervention protocols (or manuals) were requested to aid the BCT coding, if they were not included in the RCT publications or as additional materials.

#### Comparators

Studies comparing interventions targeting self-directed health behaviours (i.e., physical activity and/or weight loss) to usual/standard (e.g., advice from their health care provider).

#### Outcomes

The rationale for including these outcomes is based on a consensus study (including 26 experts from 13 countries) which identified core outcomes for multimorbidity intervention studies [26]. This consensus highlighted the importance of selective outcome measures relevant for people with multimorbidity to help create a body of evidence for people with multimorbidity as opposed to people with a single condition. Additionally, the choice of adding weight loss as an outcome was supported by the patient partner of MOBILIZE (the study within which the review was conducted) with whom we discussed the systematic review and outcome measures included. We included studies assessing at least one of the following outcomes:

- Physical activity (objectively measured or self-reported), weight loss, physical function (objectively measured or self-reported); health-related quality of life and depression symptoms.
- Physical activity and weight loss were the prespecified primary outcomes [24]. These outcomes were included to adhere to recommendations from a consensus paper on which outcomes to use in intervention studies, including people with multimorbidity [26]. The choice of these outcomes was also supported by the patient partners of MOBILIZE who were invited to comment on the current systematic review and the outcome measures included.

#### Exclusion Criteria

We excluded interventions not targeting physical activity, those targeting health care professionals, and those solely delivered via a digital solution (i.e., eHealth) to avoid repetition of an on-going systematic review (https://osf.io/5nwyr/). RCTs published in languages other than English, Scandinavian, and Italian and RCTs including less than 100% of participants with at least two of the chronic conditions of interest for this systematic review were also excluded.

#### Literature Search

We searched for studies in the Cochrane Database of Systematic Reviews, MEDLINE via PubMed, EMBASE via Ovid, CINAHL (including preCINAHL) via EBSCO, and the World Health Organization International Clinical Trials Registry Platform (ICTRP). The search was performed on June 19th, 2020, and was adapted from two reviews of the MOBILIZE project [27] https://osf.io/eszb7/ (Additional file 1). The search was restricted to studies published after 2000 given that RCTs published before this date would likely not reflect the interventions, and behaviour change techniques used, provided currently. Additionally, the reference lists of the included articles and citation tracking were also performed using Web of Science. We also screened the latest Cochrane systematic review reference lists, including people with multimorbidity [17]. Furthermore, we screened for completed trials in the World Health Organization’s ICTRP http://apps.who.int/trialsearch/ comprising the 16 primary registries of the WHO registry network and ClinicalTrials.gov. We additionally searched Web of Science for studies citing the RCTs included in this systematic review (citations tracking).

#### Search Method and Study Selection

The search strategy was developed for MEDLINE and was customised for EMBASE, CINAHL, and CENTRAL (Additional file Table 1). All terms were searched both as keywords (Mesh) and as text words in title and abstract, when possible. We used the Cochrane sensitive search strategy for identifying RCTs. We have not search for unpublished studies due to the several issues related to identifying these studies [28]. The literature search results were uploaded to Covidence, and two reviewers (AB and LKH) independently screened titles and abstracts. All studies deemed eligible by at least one of the two reviewers were checked independently in full text by the same two reviewers. Disagreements between the reviewers about the inclusion of individual studies were discussed until consensus was reached. We recorded the reasons for excluding full-text RCTs. To identify multiple reports from the same study, we checked whether multiple reports from the same study were published by juxtaposing author names, treatment comparisons, sample sizes, and outcomes. If multiple reports of the same studies provide different study characteristics such as the number of participants and presence of chronic conditions, we used the primary publication.

**Table 1:** Study, participant, intervention and outcome characteristics of the included studies.

**Author, year, and study acronym**	**Country, study design, and setting**	**Condition type, prevalence**	**Condition diagnosis and severity at baseline**	**Age (mean), gender, and BMI (mean)**	**Intervention characteristics**	Duration (minutes), frequency, length, and adherence ((number of intervention sessions attended/number of total sessions available)*100) to the behavioural intervention	**Outcomes and (outcome measure)**
Koukouvou et al. [50]	Greece, 2-arm RCT, outpatient fitness centres	D (100%) HF (100%) H (12%)	D (BDI = 18, mild to moderate) HF (NYHA class II to III) H (SBP ≥ 140 DBP ≥ 90)	52 years 0% female BMI 28	Exercise therapy	60 min, 4 times per week for 26 weeks at a moderate intensity. Adherence 78%.	Weight (BMI)* HRQoL (QLI) Depression (BDI)
Kulcu et al. [49]	Turkey, 2-arm RCT, cardiopulmonary rehabilitation clinic	D (100%) HF (100%)	D (BDI = 19, moderate to severe) HF (NYHA class II to III)	59 years 27% female	Exercise therapy	60 min, 3 times per week for 8 weeks at a moderate intensity. Adherence NR.	HRQoL (HQOL) Depression (BDI)
Katon et al. [43]	USA, 2-arm RCT, primary care clinics	D (100%) T2DM (100%) Coronary heart disease (27%)	D (PHQ-9 = 14, moderate) T2DM (glycated haemoglobin = %8) Coronary heart disease (MI, IHD angina pectoris)	57 years 52% female BMI 37	Self-care + pharmacotherapy	Clinic visits every 2 to 3 weeks, for 52 weeks. Adherence NR.	PA (adherence to exercise plan ≥ 2 days per week) Depression (SCL-20) HRQoL (QoL 10 scale)
Gary et al. [42]	USA, 4-arm RCT, home-based	D (100%) HF (100%) H (88%) T2DM (29%)	D (BDI-II = 20, moderate) HF (NYHA class II to III) H (SBP ≥ 140 DBP ≥ 90) T2DM (NA)	66 years 57% female	(1) Exercise therapy (2) CBT and exercise therapy (3) CBT	45 min, 3 times per week for 12 weeks at a moderate intensity. Adherence 82%.	HRQoL (MLHFQ) PF (6MWT) Depression (HADS-D)
Piette et al. [40]	USA, 2-arm RCT, telephone based + home-based	D (100%) T2DM (100%)	D (BDI = 26, moderate to severe) T2DM (Hba1c (%) = 7.6%)	56 years 51% female 38 BMI	CBT + walking program	12 weekly sessions followed by nine monthly booster sessions in 52 weeks. Adherence CBT 64%.	PA (step counts) HRQoL (SF-12 pcs) PF (SF-12 PF) Depression (BDI)
Åsa et al. [47]	Sweden, 2-arm RCT, outpatient centre-based	HF (100%) T2DM (100%)	HF (NYHA II–III) T2DM (Hba1c (%) = 7.4)	61 years 20% female BMI 29	Exercise therapy	45 min, 3 times a week for 8 weeks at a low to moderate intensity. Adherence 92%.	HRQoL (MLHFQ) PF (6MWT)
Lynch et al. [44]	USA, 2-arm RCT, community-based	H (100%) T2DM (100%)	H (medication usage) T2DM (medication usage)	54 years 67% female 36 BMI	Self-management	120 min, 18 sessions in 26 weeks + weekly telephone calls. Adherence NR.	Self-reported physical activity (CHAMP) Weight loss (kg)
Dunbar et al. [41]	USA, 2-arm RCT, home-based and clinic-based	HF (100%) T2DM (100%)	HF (NYHA II–IV) T2DM (Hba1c (%) = 8)	57 years, 34% female, BMI 37	Integrated self-care Intervention + usual care	One individualised counselling session with family members + one home visit by the research nurse + four telephone calls + one visit clinic. Duration 17 weeks. Adherence NR.	PA (CHAMP)
Keihani et al. [48]	Iran, 2-arm RCT, institute of cardiovascular rehabilitation in Isfahan	D (100%) HF (100%)	D (BDI = 43, severe) HF (ejection fraction equal to or less than 35%)	61 years 40% female BMI 29	Exercise therapy	60 min, 3 times per week for 8 weeks at a moderate intensity. Adherence NR.	PF (SF-36 PF) Depression (BDI-D)
Freedland et al. [38]	USA, 2-arm RCT, academic centre	D (100%) HF (100%) H (72%) T2DM (38%) COPD (18%)	D (BDI-II = 30, severe) HF (NYHA class I to III)	56 years, 46% female, 36 BMI	CBT + usual care	60 min, once per week for 26 weeks and 4 telephone calls from week 26 to 52.	PA (actigraphy 7-d average activity) PF (6MWT) Depression BDI-II) Weight loss (BMI)*
Pibernik-Okanović et al. [46]	Croatia, 3-arm RCT, tertiary diabetes clinic	D (100%) T2DM (100%)	D (CES-D = 30, severe) T2DM (Hba1c (%) = 7.3)	66 years 54% female BMI 30	(1)Exercise therapy (2)Psychoeducation	75 min, for once a week for 6 weeks. Adherence NR.	HRQoL (SF-12) Depression (CES-D)
Huang et al. [39]	Taiwan, 2-arm RCT, clinic	D (100%) T2DM (100%)	D (CES-D ≥ 16, moderate) T2DM (Hba1c (%) = 7.7)	54 years, 52% female, BMI 26	CBT + motivational enhancement therapy + usual care	80 min, once a week for 12 weeks (4 weeks of motivational enhancement therapy and 8 CBT sessions)	Weight loss (BMI) HRQoL (SF-12 pcs) Depression (CES-D)
Schneider et al. [45]	USA, 2-arm RCT, University of Massachusetts Medical School’s	D (100%) T2DM (100%)	D (BDI-II = 20, moderate) T2DM (Hba1c (%) = 7.9)	53 years 100% female BMI 31	Exercise therapy	90 min, 2 times per week for 12 weeks at a moderate intensity. Adherence 51%.	Depression symptoms (BDI-II)
de Groot et al. [37] (ACTIVE II)	USA, 2-arm RCT, community fitness centres	D (100%) T2DM (100%)	D (BDI-II = 25, moderate) T2DM (Hba1c (%) ≥ 7%)	56 years 77% female	(1) Exercise therapy (2) Exercise therapy and CBT (3) CBT	50 min (10 min warm up and 10 min cool down) 2 times per week for 12 weeks at a moderate intensity	Depression (BDI-II) HRQoL (SF-12 pcs) PF (6MWT)

*BDI* Beck depression inventory, *BDI-II* Beck depression inventory II, *BMI* body mass index, *CES-D* Center for Epidemiologic Studies Depression Scale, *COPD* chronic obstructive pulmonary disease, *D* depression, *EuroQol-VAS* EQ quality of life visual analogue scale, *GDS* geriatric depression scale, *H* hypertension, *HF* heart failure, *HADS-D* hospital and anxiety depression scale for depression (D), *HbA1c* haemoglobin A1c, *HQOL* Hacettepe Quality of Life Questionnaire, *HRQoL* health-related quality of life, *MLHFQ* Minnesota Living with Heart Failure Questionnaire, *PF* physical function, *6MWT* six-minute walking test, *RCT* randomised controlled trial, *PA* physical activity, *PHQ-9* Patient Health Questionnaire-9, *QLI* Quality of Life Index, *SCL-20* Symptom Checklist–20, *SF-12* 12-item Short Form Health Survey, *SF-36* 36-item Long Form Health Survey, *T2DM* type 2 diabetes mellitus

*Data retrieved upon request from the authors of the study

#### Data Collection

The following data were extracted from end-treatment follow-ups (immediately after the intervention) and follow-ups as close to 12 months as possible.

Study characteristics: location of the trial, number of patients allocated to the exercise and comparator groups, respectively, number of patients in the intention to treat (ITT), and per protocol analysis, in the intervention and comparator groups, respectively.

Participant characteristics: age, proportion of female, body mass index (BMI), baseline severity and diagnosis of the conditions, and number, type, and frequency of other conditions ethnicity, and socioeconomic status (SES) (i.e., studies were labelled as ‘low SES’ when most of the participants were described as having low education levels, low income, being unemployed, homeless, receiving government benefits, in prison, or sample was labelled as ‘low SES’ in the included RCTs) [29].

Intervention and comparator characteristics using the Template for Intervention Description and Replication (TIDieR) checklist [30]. This includes 12 items that are brief name of the intervention, why (rationale, theory, or goal of the elements essential to the intervention), what (materials used in the interventions), what (procedure activities and/or processes used in the intervention), who provided the intervention (e.g., exercise physiologist), how (modes of delivery), where (type(s) of location(s) where the intervention occurred), when and how much (number of times the intervention was delivered), tailoring (if the intervention was planned to be personalised, titrated, or adapted, then describe what, why, when, and how), modifications (if the intervention was modified during the study), describe the changes (what, why, when, and how), how well (planned adherence and fidelity), and how well (actual adherence and fidelity).

Outcome characteristics: time points assessed and the magnitude of objectively and subjectively measured changes (e.g., change in physical activity). To avoid multiplicity, we used a hierarchy of selection rules for the outcomes.

#### Outcome Selection Hierarchy

We prioritised extracting generic outcome measures, rather than disease-specific, that were most reported (e.g., 6MWT) for each outcome domain (e.g., physical function). This method has been previously applied for people with multimorbidity [15] and was guided by a scoping review mapping the behaviour change techniques used in patient-centred interventions for people with multimorbidity (https://osf.io/svt35/).

For objectively measured physical activity, we prioritised: (1) accelerometer measures (e.g., daily time spent in moderate to vigorous physical activity); (2) pedometer (e.g., outcomes such as step counts); and (3) any other outcome measure related to objectively measured physical activity.

For subjectively measured physical activity, we prioritised: (1) the Global Physical Activity Questionnaire; (2) the Physical Activity Scale for the Elderly (PASE) Questionnaire; (3) the International Physical Activity Questionnaires (IPAQ) long, short form, and modified versions (e.g., for the elderly); and (4) any other outcome measure related to subjectively measured physical activity.

For weight loss outcome measures, we prioritised: (1) change in body mass index; (2) change in weight; and (3) any other measure.

For health-related quality of life, we prioritised: (1) the EQ-5D questionnaire, (2) any other general health-related quality of life questionnaires (e.g., the 36-item Short-Form Health Survey physical component summary), and (3) disease-specific health-related quality of life questionnaires (e.g., The Minnesota Living with Heart Failure Questionnaire).

For objectively measured physical function, we prioritised: (1) the 6-minute walking test, (2) Incremental Shuttle Walking Test, and (3) any other outcome measure related to daily function (e.g., chair stand test).

For self-reported physical function, we prioritised: (1) the SF-36 Physical Function subscale, (2) the SF-36 Role Function subscale, and (3) any other self-reported measure of physical function.

For continuous outcomes, we extracted the number of participants, mean and standard deviation, standard error or 95% confidence interval, *P* value, or other methods recommended by the Cochrane Collaboration [21]. If the data could not be extracted from the published studies, we emailed the corresponding author a checklist including the data we aimed to obtain. If the email we sent bounced back, we contacted the second author and so forth. After 3 days, we sent a reminder. After 7 days of the first email, we re-sent the email to the corresponding and last authors. A second reminder followed 10 days after the first email. We considered the data as missing after not receiving any communication from the authors 15 days after sending the first email.

### Risk of Bias Assessment and Overall Evaluation of the Quality of the Evidence

The two reviewers (AB and LKH) independently assessed the internal validity of all included studies using the Cochrane ‘Risk of Bias Tool’ (version 2.0). This tool includes the following domains: (1) bias arising from the randomisation process; (2) bias due to deviations from the intended interventions; (3) bias due to missing outcome data; (4) bias in measurement of the outcome; and (5) bias in selection of the reported result. Within each domain, the two reviewers answered one or more signalling questions (e.g., Was the allocation sequence random? Were participants aware of their assigned intervention during the trial?) which led to judgments of ‘low risk of bias’, ‘some concerns’, or ‘high risk of bias’. The judgments within each domain lead to an overall risk-of-bias judgment for the assessed outcome [21]. Disagreements were resolved through discussion until consensus was reached. The overall quality of evidence for the estimates was evaluated using the GRADE (Grading of Recommendations Assessment, Development and Evaluation) approach [31]. The GRADE is a systematic approach to rate the quality of evidence across studies for specific outcomes. It is based on five domains that involve the methodological flaws of the studies (i.e., risk of bias), the heterogeneity of results across studies (i.e., inconsistency), the generalisability of the findings to the target population (i.e., indirectness), the precision of the estimates, and the risk of publication bias [31].

### Synthesis of Results

We performed meta-analysis to assess the average effect of behavioural interventions on the outcomes of interest using a random-effects model as heterogeneity was expected due to differences in interventions, outcome measures, etc. Statistical heterogeneity was examined as between-study variance and calculated as the *I*-squared statistic measuring the proportion of variation in the combined estimates due to between-study variance. An *I*-squared value of 0% indicates no statistical heterogenity between the results of individual studies, and an *I*-squared value of 100% indicates maximal statistical heterogentity. Standardised mean differences (SMDs) with 95% CIs were calculated for outcome measures of continuous data but measured in different ways (e.g., all studies measured physical activity, but they use different objective tools) and adjusted to Hedges’ *g*. On the other hand, for outcomes of continuous data measured in the same way (e.g., all studies measured weight loss assessing the BMI), the mean differences (MDs) with 95% CIs were calculated. The magnitude of the effect size of the pooled SMD was interpreted as 0.2 representing a small effect, 0.5 a moderate effect, and 0.8 a large effect [21]. For outcome measures where a meta-analysis was not possible, a narrative data synthesis of the results from individual studies was performed in line with the guidance from the Cochrane Handbook [21]. When several intervention groups were compared to one control group, the number of participants in the control group was divided by the number of intervention groups, and each was analysed as a separate study comparison [21]. Meta-analyses were performed in STATA (V.17.0) using the ‘meta’ command.

### Meta-regression Analyses and Effectiveness Ratio

Prespecified meta-regression analyses [24] were performed to explain heterogeneity by exploring the association of different BCTs, participants, studies, and intervention characteristics with effect estimates. Given the explorative nature of such analyses, the most commonly reported (at least in 10 studies as per Cochrane Handbook guidelines) patient, intervention, and study characteristics were chosen as moderators, but no prior hypotheses were made on the possible associations. However, since too few studies were included in the meta-analyses for physical activity and weight loss, we did not perform meta-regression analysis for these outcomes according to the Cochrane Handbook [21]. Instead, we investigated the association between BCTs and these outcomes narratively, by calculating the effectiveness ratios (i.e., the ratio of the number of times each BCT was used in an effective trial divided by the number of times the BCT was used in all trials). This was not prespecified. An effective trial was defined as a trial reporting a statistically significant between-group difference (*P* < 0.05) or a SMD ± 0.2 [21] in favour of the intervention group. This method has been used in published systematic reviews of similar topics [32–34], is deemed acceptable by the Cochrane Handbook [35], and was only used when at least three study comparisons were available to avoid overinterpreting the results.

### Sensitivity and Additional Analyses not Prespecified

We performed two sensitivity analyses to explore the robustness of the findings. First, given that physical activity and physical function are on the same continuum in the International Classification of Functioning, Disability and Health contextualisation, they were pooled together in one meta-analysis [36]. Second, the meta-analysis on health-related quality of life was repeated, including the mental component scores instead of the physical component scores of the SF-12 [37–40]. This was done due to the fact that both the physical and mental component scores of the SF-12 can be used to measure health-related quality of life. Furthermore, as the majority of the studies included patients with depression and targeted depression symptoms in addition to lifestyle behaviours, we also assessed the effect of behavioural intervention on depression symptoms.

### Patients’ Involvement

The MOBILIZE project is committed to patient involvement and has so far included patients living with multimorbidity in all aspects of the decision-making process in the project. Their experiences, needs, and preferences play an important role in developing a novel intervention (Collaborate level on the IAP2 Spectrum of Public Participation). For this systematic review, two patient partners of the MOBILIZE project were introduced to the review and provided feedback on what outcomes to include, before starting the review.

## Results

### Study Selection and Characteristics

The search identified a total of 1226 unique publications, of which 95 individual RCTs were identified and full texts screened for potential eligibility. Ultimately, we included 14 studies (see Additional file 2 for an overview). The included studies were conducted in 7 countries: USA [37, 38, 40–45], Croatia [46], Sweden [47], Iran [48], Turkey [49], Greece [50], and Taiwan [39] and were published from 2010 to 2019. The study authors of two studies [38, 50] were contacted for clarification on outcome data and for requesting additional data. Both authors replied, clarified, and provided the data requested. The characteristics of the included studies are reported in Table 1.

### Participant Characteristics

The overall mean age of the participants (*n* = 1,378) included in the studies was 58.1 (SD ± 4.7), 50.9% were female, and mean BMI was 32.5 (SD ± 4.6). The most common combination of conditions reported was type 2 diabetes and depression in 6 studies [37, 39, 40, 43, 45, 46], depression and heart failure in 5 studies [38, 42, 48–50], type 2 diabetes and heart failure in 2 studies [41, 47], and hypertension and type 2 diabetes in one study [44].

### Intervention and Comparator Group Characteristics

All the interventions targeted lifestyle behaviours, including physical activity and healthy diet. The interventions were multifaceted and, in addition to usual care (e.g., counselling from their health care provider), the most commonly used components were exercise therapy in 8 studies [37, 42, 45–50], cognitive behavioural therapy (CBT) in 4 studies [37–39, 42], patient education in 3 studies [37, 42, 46], self-care in 2 studies [41, 43], and motivation enhancement therapy [39], pharmacology [43], and behavioural activation [45] in one study. Exercise together with patient education and CBT or behavioural activation was used in 3 studies [37, 42, 45]. The comparator groups included in meta-analyses were usual care (Table 1). Therefore, when several intervention groups were included in an RCT, the between-group difference was reported for all the interventions versus a comparator group. For example, when a study had two intervention groups (e.g., Exercise and CBT) and one comparator group (Usual care), we compared ‘Exercise’ versus ‘Usual care’ and ‘CBT’ versus ‘Usual care’, and reported the results as two separate study comparisons. This procedure is in accordance with the Cochrane Handbook [21]. The BCTs used in the included studies to target lifestyle behaviours such as physical activity and weight loss are reported in Additional file 3. Overall, the BCTs most commonly used were ‘Instructions on how to perform the behaviour’ (BCT 4.1) in all the studies but one [43], ‘Social support unspecified’ (BCT 3.1) in 11 studies [37–39, 41–45, 48, 49], and ‘action planning’ (BCT 1.4) in 9 studies [37, 38, 40, 42, 45, 47–50]. The clusters of BCTs most commonly used were ‘Goals and planning’ and ‘Feedback and monitoring’ which were present 27 times in the 14 included studies.

### Outcome Characteristics

Physical activity was reported in 8 studies [38, 40, 41, 43–45, 49, 50], of which 5 used an objective assessment (e.g., accelerometer) [38, 40, 45, 49, 50] and 3 a self-reported tool [41, 43, 44]. Weight loss was reported in 6 studies [37–39, 44, 45, 50] of which 5 studies reported data about the BMI of the participants and one as kg [44]. Physical function was reported in 7 studies [37, 38, 40–42, 47, 48] of which 5 studies used an objective assessment (i.e., the 6 minutes walking test) [37, 38, 41, 42, 47] and two used a self-reported tool (i.e., the SF-12) [40, 48]. Health-related quality of life was reported in 10 studies [37–43, 47, 49, 50]. Characteristics of the outcome measures are reported in Table 1.

### Effect of Behavioural Interventions on Physical Activity

**Fig. 1:**
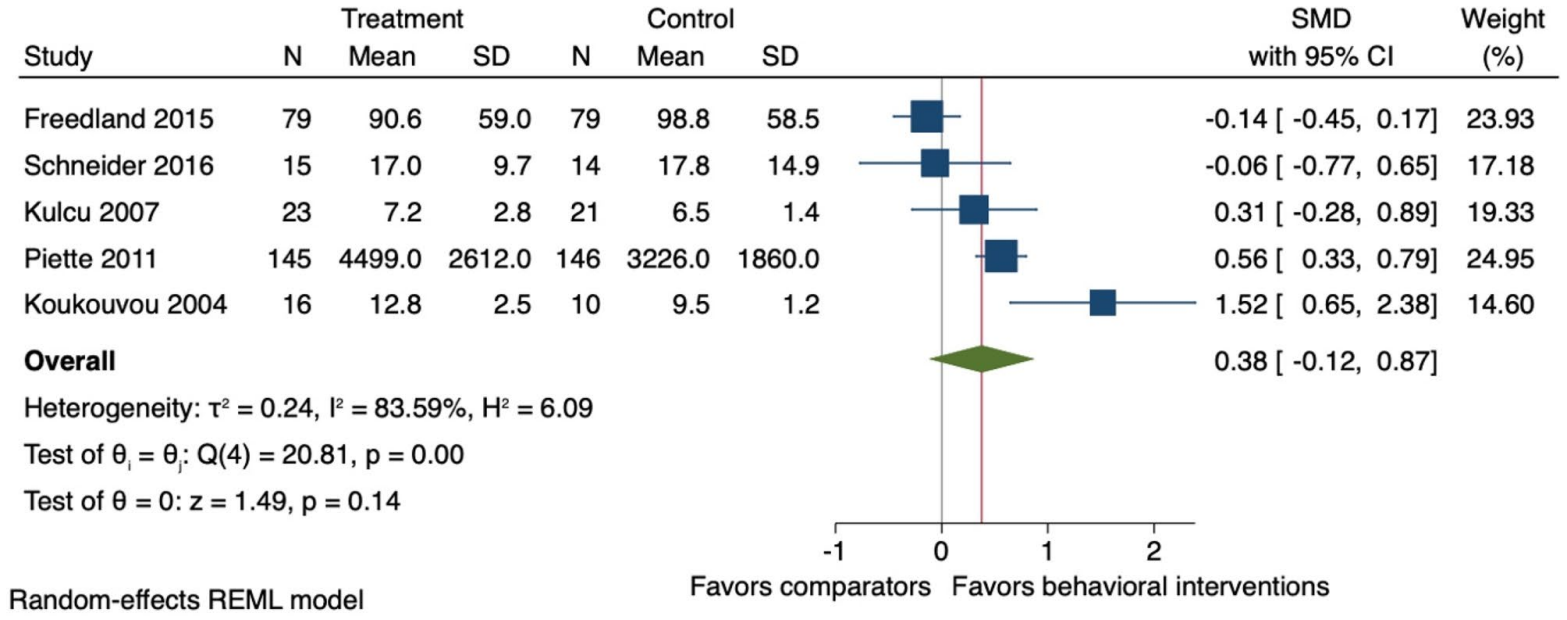
Forest plot for the effect of behavioural interventions compared to a usual care comparator group on objectively measured physical activity. SMD, standardised mean difference; 95% CI, 95% confidence interval

Five studies were included in the meta-analysis on physical activity. At the end-of-treatment follow-ups (mean 16 weeks (SD ± 4)), on average behavioural interventions appeared to have little effect on objectively measured physical activity (*k* = 5; *n* = 548; SMD 0.38, 95% CI −0.12 to 0.87; *I*^2^ = 83.6%) (Fig. 1); however, the evidence is uncertain. Only one study [45] reported data on long-term follow-up (24 weeks post randomisation), showing no difference on objectively measured physical activity between the intervention and comparator group (*k* = 1; *n* = 29; SMD 0.13, 95% CI −0.58 to 0.84).

Three studies assessed self-reported physical activity [41, 43, 44]. The results of these three studies were summarised narratively as no meta-analysis was deemed eligible due to large differences in reporting of the self-reported physical activity outcome measures. Overall, these three studies reported that the participants in the intervention groups were more physically active than the participants in the control groups at the end-treatment follow-up (mean 33 weeks, SD ± 16). One study [41] reported that the percentage of participants physically active (i.e., having a Community Healthy Activities Model Program for Seniors (CHAMPS) questionnaire score > 6) was 74.5% in the intervention group and 59.5% in the comparator group. Another study [43] reported that the percentage of participants physically active (two or more times per week) was 68.5% in the intervention group and 32.5% in the comparator group. While yet another study [44] reported that the participants in the intervention group improved their physical activity level (assessed with the CHAMPS questionnaires) more than the comparator group (*P* < 0.05).

### BCTs Associated with Physical Activity (Objectively Measured and Self-Reported)

**Fig. 2:**
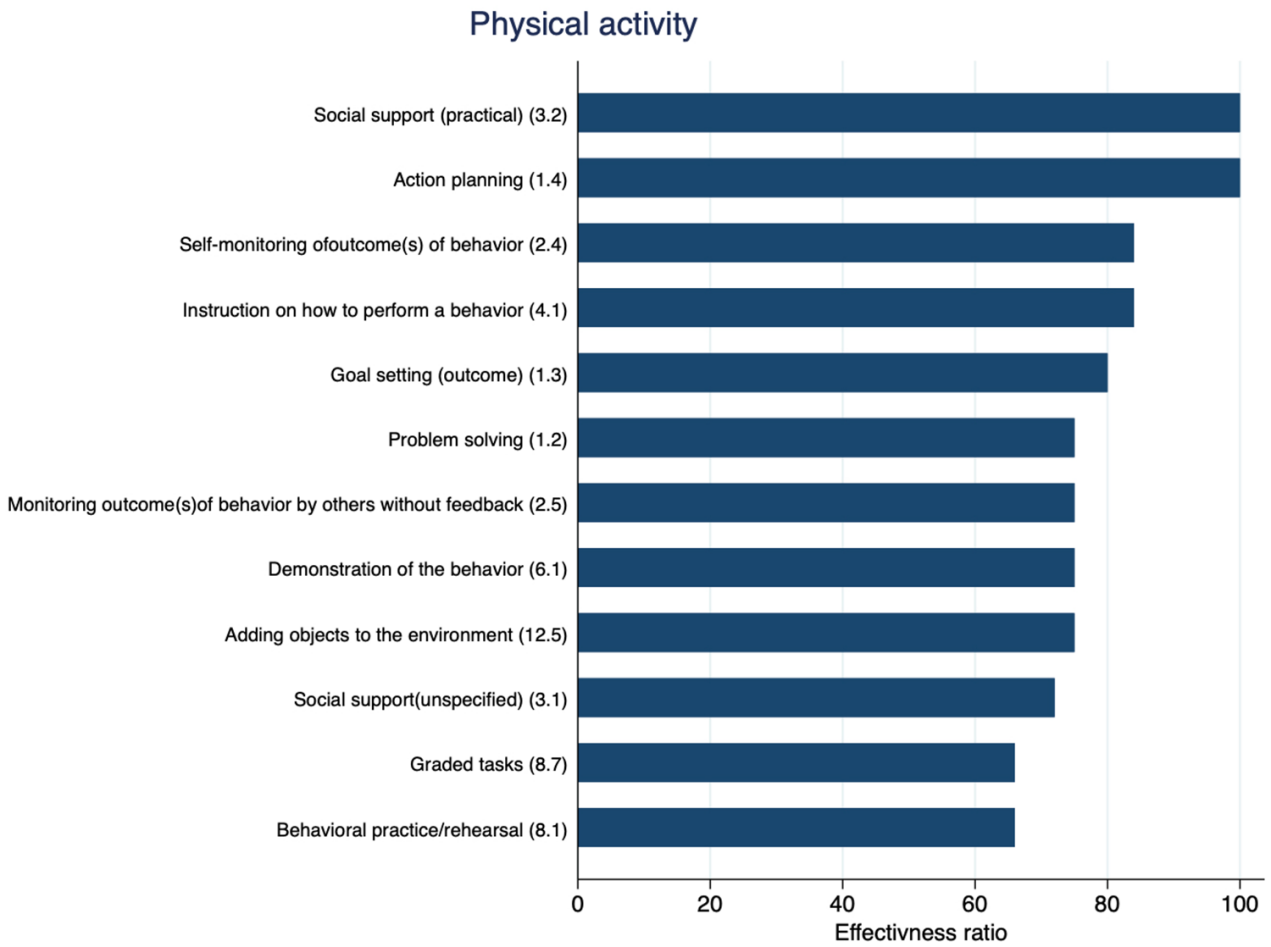
Effectiveness ratio of BCTs in behavioural randomised controlled trials including people with multimorbidity. Effectiveness ratio (*x*-axis) = number of times each BCT (*y*-axis) was used in an effective trial divided by the number of times they were a component of all studies using the BCT; the higher the ratio, the more often the BCT was found effective out of the total number of studies included; *x*-axis = effectiveness ratio, *y*-axis = BCTs

Overall, 12 BCTs were reported in at least 3 study comparisons at the end-treatment follow-up, and effectiveness ratios were calculated. Ten of the 12 BCTs tested had an effectiveness ratio of more than or equal to 75%, with the BCT 3.2 ‘social support (practical)’ and BCT 1.4 ‘action planning’ having an effectiveness ratio of 100% (Fig. 2). At the follow-up closest to 12 months, we were unable to calculate effectiveness ratios due to insufficient data. Additional file 4 reports the raw data for calculating the effectiveness ratios.

### Effect of Behavioural Interventions on Weight Loss

**Fig. 3:**
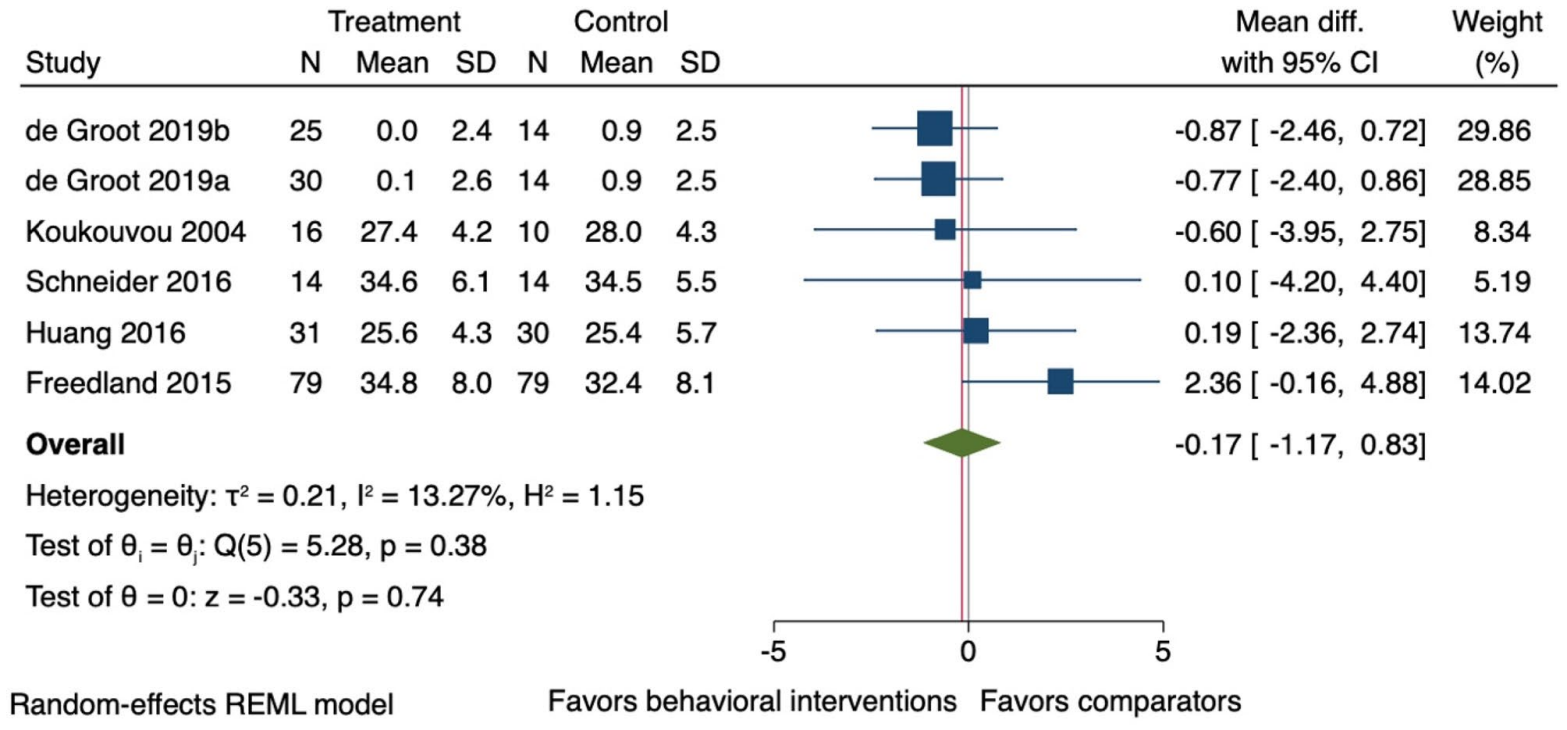
Forest plot for the effect of behavioural interventions compared to a usual care comparator group on weight loss (body mass index). 95% CI, 95% confidence interval. ^a,b^ = two separate study comparisons from the same study

Five studies were included in the meta-analysis on weight loss [37–39, 45, 50] with end-of-treatment follow-ups (mean 18 weeks (SD ± 7)). It is uncertain whether on average behavioural interventions had an effect on weight loss (*k* = 6; *n* = 356; BMI mean difference −0.17, 95% CI −1.17 to 0.83: *I*^2^ = 13.3%) (Fig. 3). The study not included in a meta-analysis reported that the intervention group lost 1.8 kg (95% CI −4.3 to 0.8) more than the comparator group [44]. Two studies were included in the meta-analysis with long-term follow-ups (24 months post randomisation) [39, 45] showing uncertainty for the effect of behavioural interventions on weight loss (*k* = 2; *n* = 86; BMI mean difference −0.54, 95% CI −2.70 to 1.62; *I*^2^ = 0.0%) (Additional file 4).

### BCTs Associated with Weight Loss

**Fig. 4:**
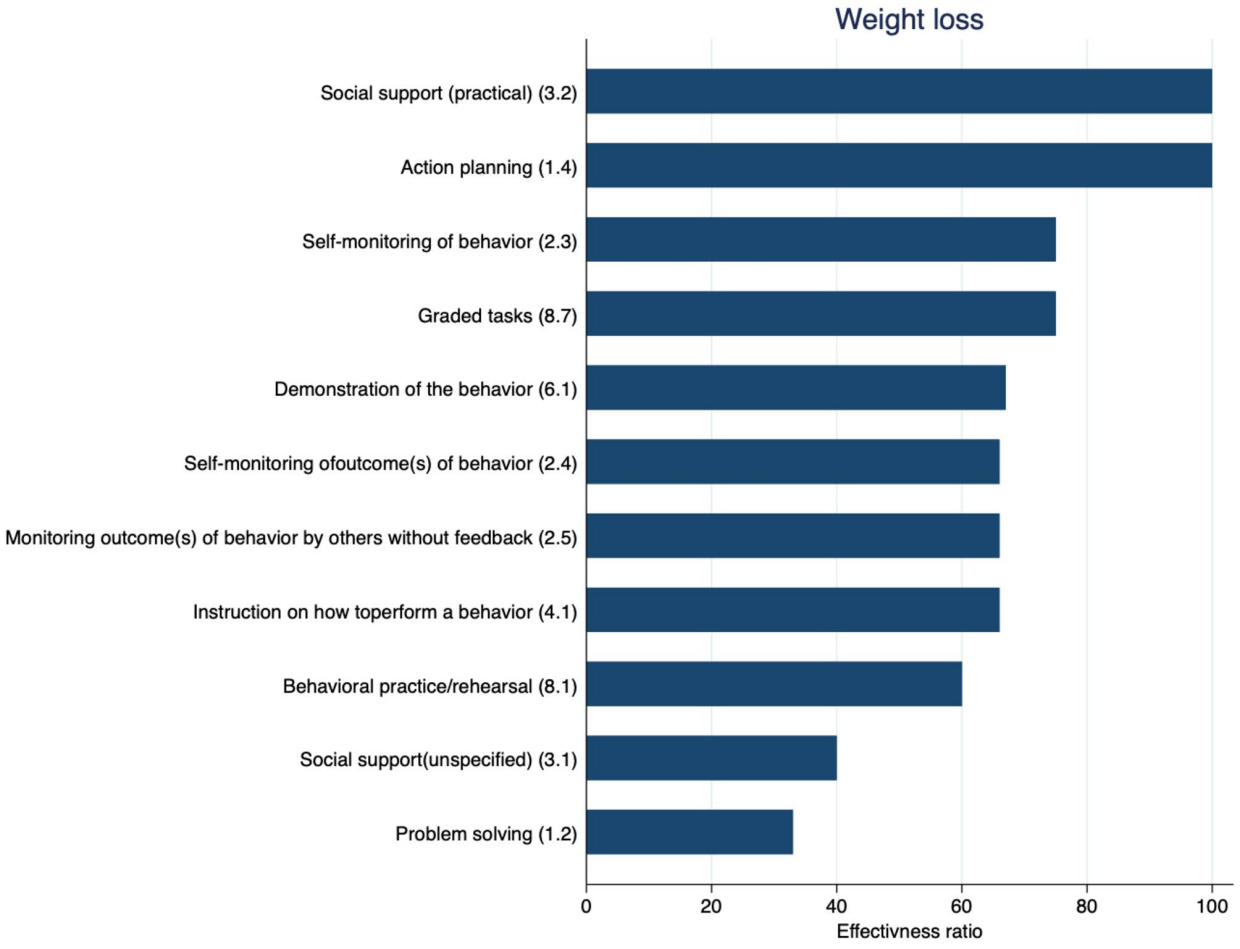
Effectiveness ratio of BCTs in behavioural randomised controlled trials including people with multimorbidity. Effectiveness ratio (*x*-axis) = number of times each BCT (*y*-axis) was used in an effective trial divided by the number of times they were a component of all studies using the BCT; the higher the ratio, the more often the BCT was found effective out of the total number of studies included; *x*-axis = effectiveness ratio, *y*-axis = BCTs

Overall, 11 BCTs were reported in at least 3 study comparisons, and effectiveness ratios were calculated. Five of the 11 BCT tested had an effectiveness ratio of more than or equal to 75%, with the BCT 3.2 ‘social support (practical)’ and BCT 1.4 ‘action planning’ having an effectiveness ratio of 100% (Fig. 4). At the follow-up closest to 12 months, we were unable to calculate effectiveness ratios due to insufficient data. Additional file 4 reports the raw data for calculating the effectiveness ratios.

### Effect of Behavioural Interventions on Health-Related Quality of Life

**Fig. 5:**
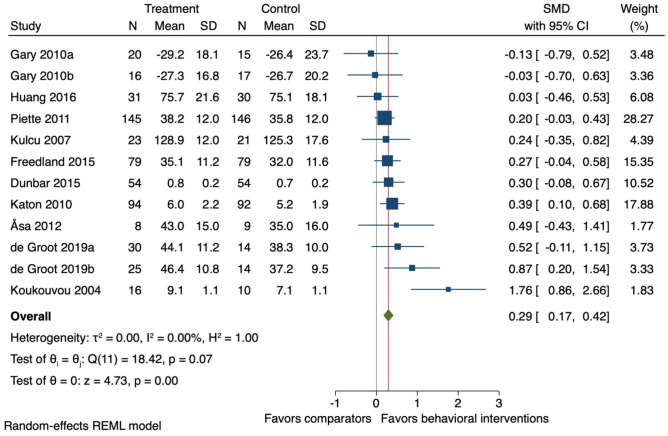
Forest plot for the effect of behavioural interventions compared to a usual care comparator group on health-related quality of life. SMD, standardised mean difference; 95% CI, 95% confidence interval. ^a,b^ = two separate study comparisons from the same study

Ten studies were included in meta-analysis on health-related quality of life at the end-treatment follow-up (mean 17 weeks (SD ± 13)). On average, behavioural interventions improved health-related quality of life (*k* = 10; *n* = 1,042; SMD 0.29, 95% CI 0.17 to 0.42: *I*^2^ = 0.0%) (Fig. 5). Three studies were included in the meta-analysis with long-term follow-ups (24 months post randomisation) [38, 39, 42] and one study was included in the narrative synthesis. Meta-analysis showed that behavioural interventions may improve health-related quality of life (*k* = 3; *n* = 233; SMD 0.20, 95% CI −0.05 to 0.46; *I*^2^ = 0.0%). However, the evidence was uncertain (Additional file 5), and the study included in the narrative synthesis showed no difference between the intervention and comparator groups [46]. We did not conduct meta-regression analyses or effectiveness ratio for health-related quality of life due to the absence of statistical heterogeneity in the meta-analysis.

### Effect of Behavioural Interventions on Physical Function

**Fig. 6:**
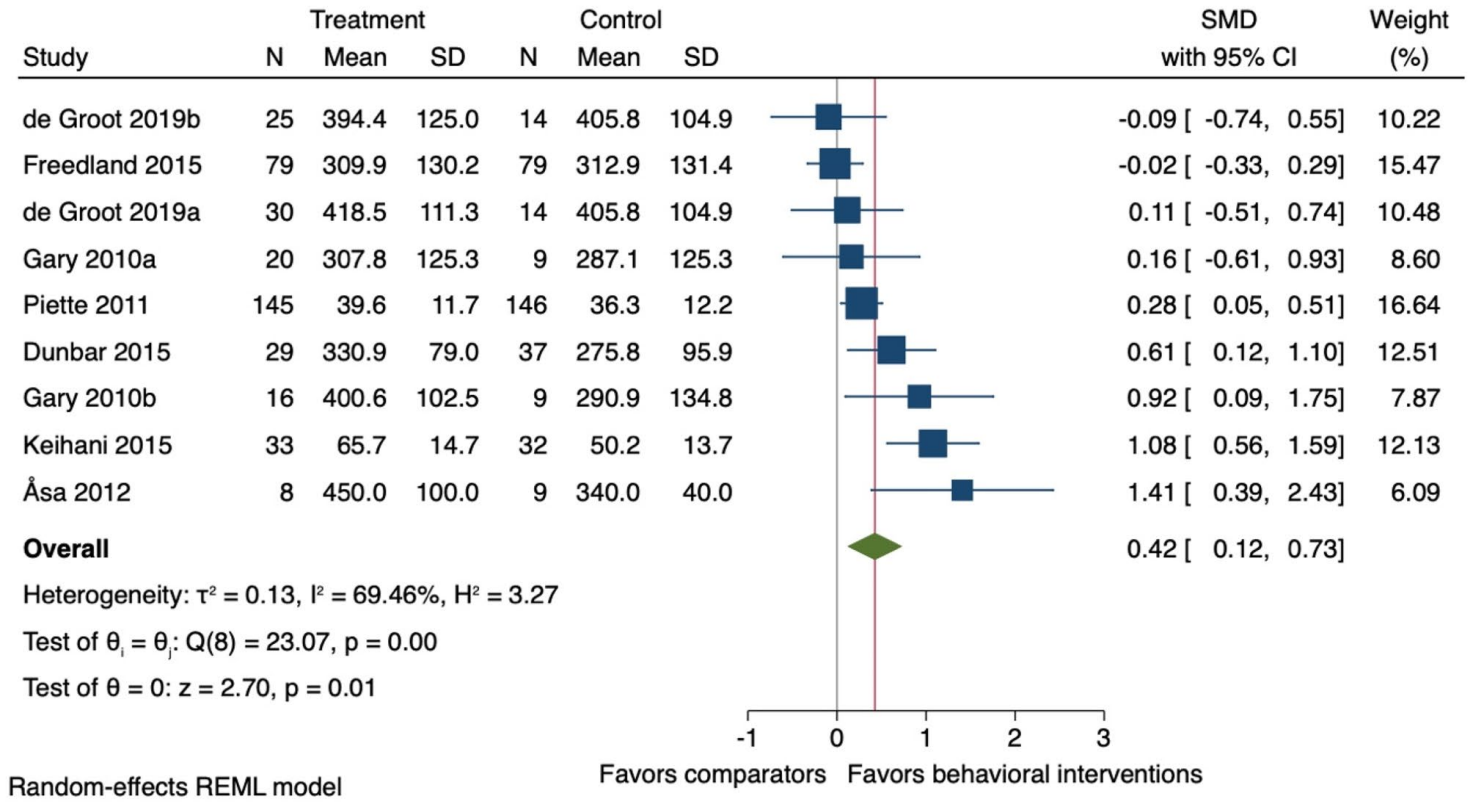
Forest plot for the effect of behavioural interventions compared to a usual care comparator group on physical function. SMD, standardised mean difference; 95% CI, 95% confidence interval. ^a,b^ = two separate study comparisons from the same study

Eight studies were included in meta-analysis for physical function at the end-of-treatment follow-up (mean 12 weeks (SD ± 5)). On average, behavioural interventions improved physical function (*k* = 8; *n* = 734; SMD 0.42, 95% CI −0.12 to 0.73: *I*^2^ = 69.5%) (Fig. 6). Meta-regression analysis showed that increasing age was associated with higher effect sizes (slope 0.07, 95% CI 0.02 to 0.13) explaining 65% (Adjusted *R*^2^) of the inconsistency of the findings. A higher proportion of female participants in the studies was associated with lower effect sizes (slope −0.02, 95% CI −0.04 to −0.01) explaining 36% (Adjusted R^2^) of the inconsistency of the findings. Meta-regression analysis also showed that studies using the BCT 2.1 ‘Monitoring of outcome of behaviour by others without feedback’ were associated with a lower improvement in physical function than studies not using this BCT. Additionally, meta-regression analysis showed that studies using a higher number of BCTs for ‘goal setting and planning’ were associated with lower effect sizes (slope −0.45, 95% CI −0.72 to −0.18) and this explained 87% of the variations in the results of the meta-analysis (Additional file 6). Finally, a subgroup analysis showed that behavioural interventions including structured exercise sessions reported a moderate improvement (*k* = 6; *n* = 219; SMD 0.56, 95% CI 0.08 to 1.04) compared to interventions without a structured exercise session (*k* = 3; *n* = 515; SMD 0.25, 95% CI −0.06 to 0.56); however, there was no statistically significant difference between the two subgroups (Additional file 7).

One study, including two study comparisons, was included in the meta-analysis with long-term follow-up (24 weeks post randomisation). The study assessed physical function with the 6 minutes walking test and showed that behavioural interventions improved physical function (mean difference in metres walked in 6 min: 74.9, 95% CI 0.01 to 149.9; *I*^2^ = 0.0%).

### Additional Analyses not Prespecified

**Fig. 7:**
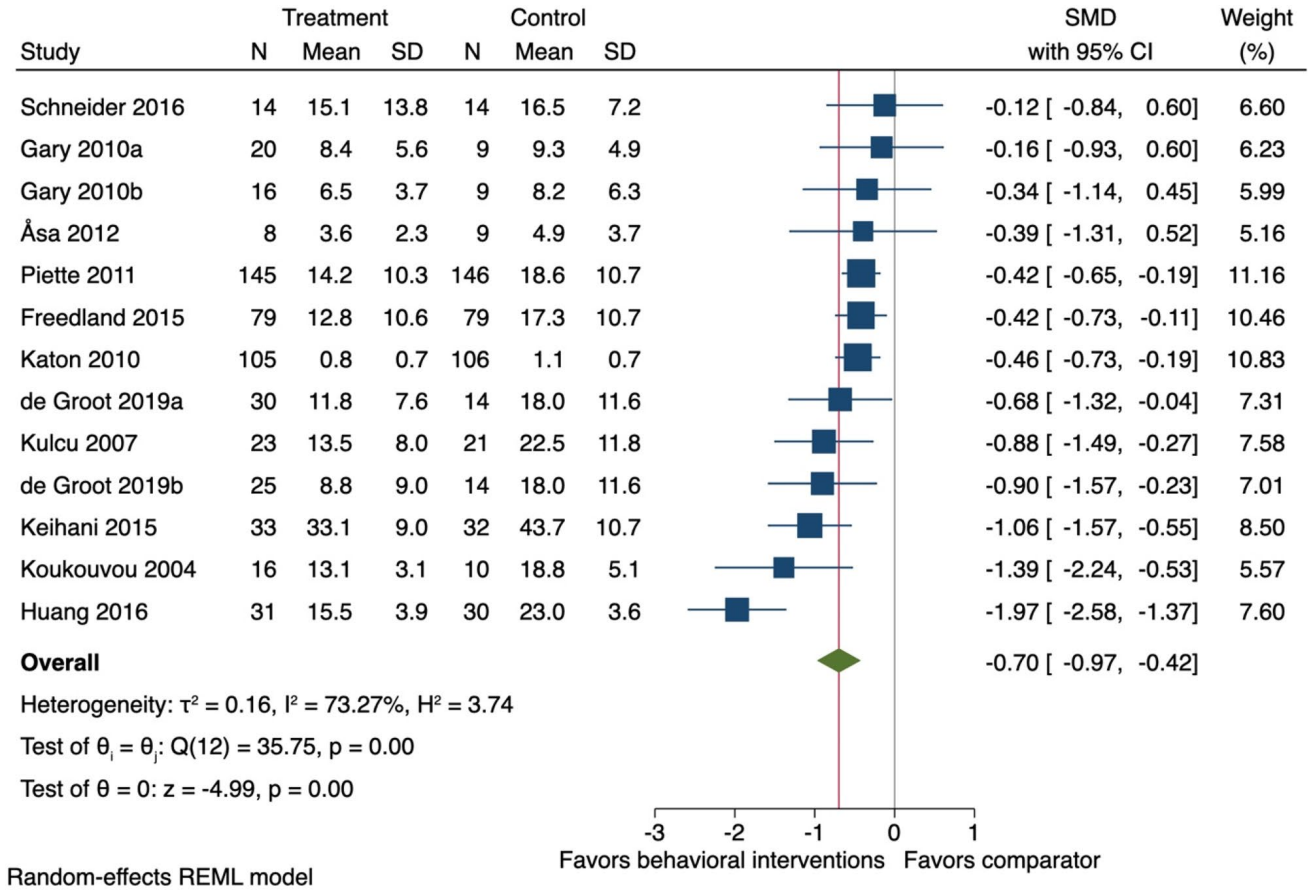
Forest plot for the effect of behavioural interventions compared to a usual care comparator group on depression symptoms. SMD, standardised mean difference; 95% CI, 95% confidence interval. ^a,b^ = two separate study comparisons from the same study

Eleven studies were included in the additional analysis investigating the effect of behavioural interventions on depression symptoms. At the end-of-treatment follow-ups (mean 14 weeks (SD ± 6)) on average, behavioural interventions reduced depression symptoms (*k* = 11; *n* = 1,038; SMD −0.70, 95% CI −0.97 to −0.42: *I*^2^ = 73.3%) (Fig. 7). At the long-term follow-up assessment, there was no effect of behavioural interventions on depression symptoms (SMD −0.38, 95% CI −1.02 to 0.26: *I*^2^ = 89.9%). Meta-regression analysis showed that studies including people with a higher BMI (slope 0.9, 95% CI 0.04 to 0.15), and studies using a higher number of BCTs for ‘goal setting and planning’ (slope 0.31, 95% CI 0.04 to 0.58) and ‘Feedback and monitoring’ (slope 0.25, 95% CI 0.02 to 0.48) were associated with a lower reduction of depression symptoms. Depression severity at baseline was not associated with depression symptoms reduction (slope 0.01, 95% CI −0.02 to 0.03).

### Sensitivity Analyses

**Fig. 8:**
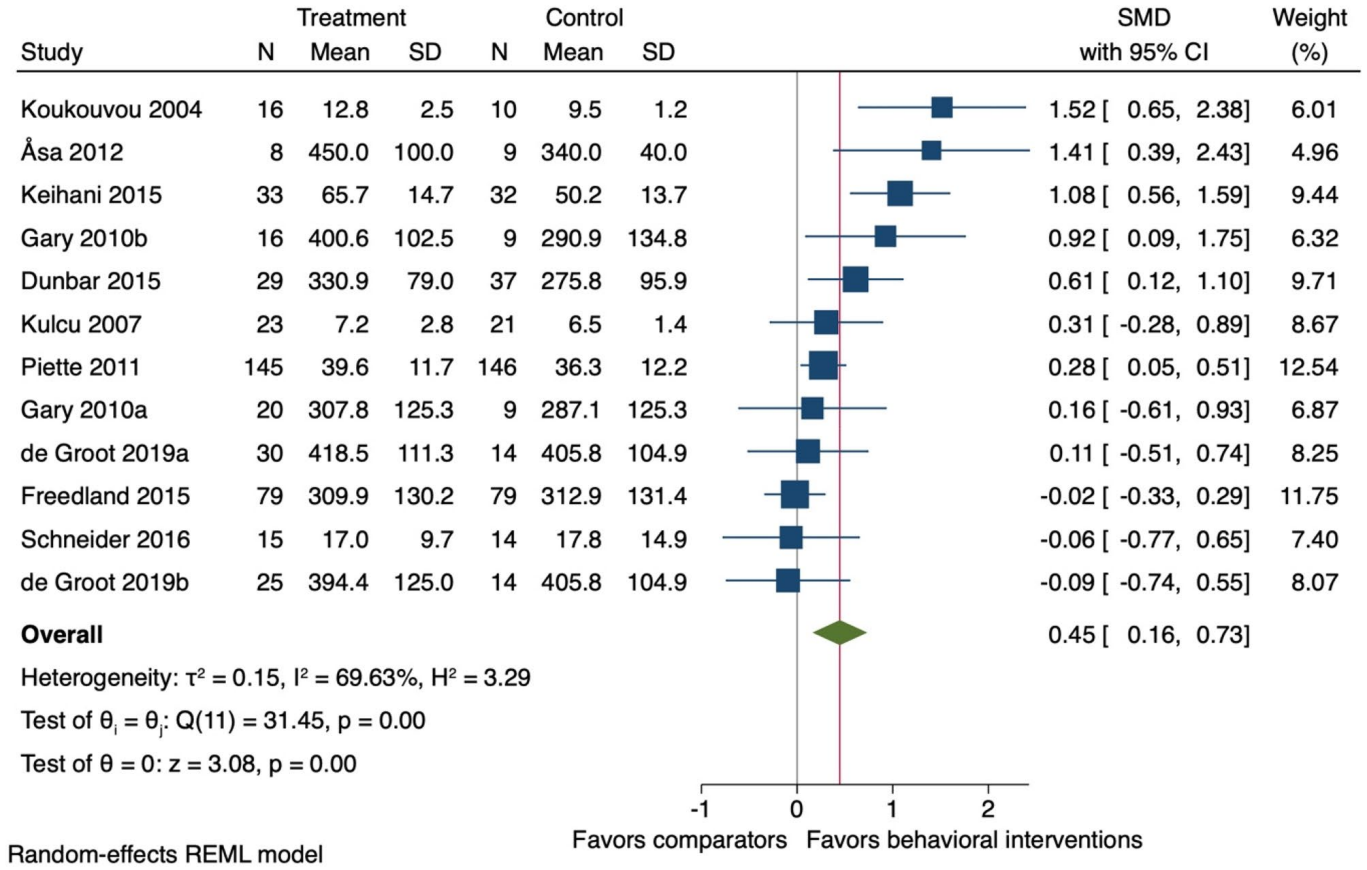
Forest plot for the effect of behavioural interventions compared to a usual care comparator group on physical activity and physical function. SMD, standardised mean difference; 95% CI, 95% confidence interval. ^a,b^ = two separate study comparisons from the same study

In the sensitivity analyses analysing physical activity and physical function together, 10 studies (12 comparisons) were included. At the end-treatment follow-ups (mean 14 weeks (SD ± 6)), behavioural interventions on average improved physical activity and physical function when combined (*k* = 12; *n* = 849; SMD 0.45, 95% CI 0.16 to 0.73: *I*^2^ = 69.6%) (Fig. 8).

**Fig. 9:**
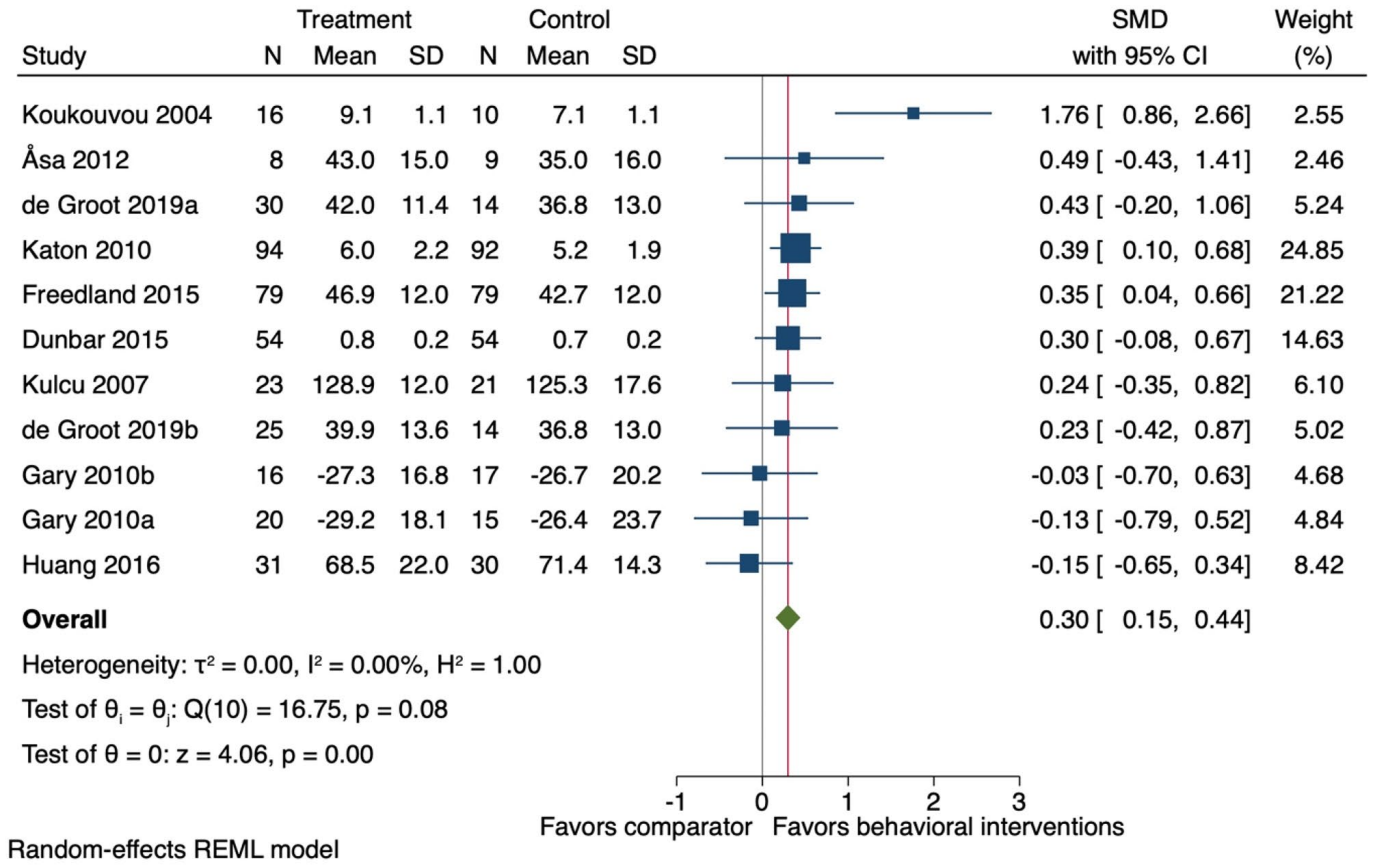
Forest plot for the effect of behavioural interventions compared to a usual care comparator group on health-related quality of life. SMD, standardised mean difference; 95% CI, 95% confidence interval. ^a,b^ = two separate study comparisons from the same study

Ten studies (11 comparisons) were included in the sensitivity for health-related quality of life (i.e., including the mental component scale data instead of the physical component score data for the studies using the SF-12). At the end-of-treatment follow-up (mean 17 weeks (SD ± 13)) on average, behavioural interventions improved health-related quality of life (*k* = 11; *n* = 754; SMD 0.30, 95% CI 0.15 to 0.44: *I*^2^ = 0.0%) (Fig. 9). These results are similar to the primary analysis results (Fig. 6).

### Risk of Bias and Overall Quality of the Evidence

The majority of the RCTs applied a proper randomisation process and reported and assessed the outcomes of interest correctly. Due to the nature of behavioural interventions, blinding of participants is challenging as patients receiving the intervention are also the outcome assessors of the patient-reported outcomes (Additional file 8). The overall quality of the evidence assessed using GRADE, including reasons for downgrading the quality of the evidence, is summarised in Table 2. Additionally, some of the included studies where possibly underpowered to detect a between-group difference due to their nature (i.e., pilot studies). However, there was no clear sign of publication bias from the visual inspection of the funnel plots suggesting no sign of small study bias (Additional file 9).

**Table 2:** Summary of findings table

Outcomes	**Risk with behavioural intervention**	No. of participants (studies)	Certainty of the evidence (GRADE)	Comments
Physical activity assessed with: objectively measured follow-up: mean 16 weeks	SMD **0.38 SD higher** (0.12 lower to 0.87 higher)	548 (5 RCTs)	⨁◯◯◯ VERY LOW^a,b,c^	Behavioural intervention may increase/have little to no effect on physical activity, at the end of the interventions, but the evidence is very uncertain. Greater short-term effects are associated with the use of the BCT 1.4 ‘action planning’ and the BCT 3.2 ‘social support (practical)’. The evidence is very uncertain for long-term effectiveness (*k* = 1).
Physical activity assessed with: self-reported follow-up: range 24 to 52 weeks	Not pooled	344 (3 RCTs)	⨁◯◯◯ VERY LOW^b,d^	The evidence is very uncertain about the effect of behavioural intervention on physical activity. The three studies included reported that the participants in the intervention groups were more physically active than the participants in the comparator groups at the end-treatment follow-up (mean 33 weeks, SD ± 16). Greater short-term effects with the use of the BCT 1.4 ‘action planning’ and the BCT 3.2 ‘social support (practical)’. No assessments were made at long-term follow-ups.
Weight loss follow-up: mean 18 weeks	MD **0.17 SD lower** (1.17 lower to 0.83 higher)	356 (5 RCTs)	⨁◯◯◯ VERY LOW^a,b,c^	The evidence is very uncertain about the effect of behavioural intervention on weight loss. One study not included in meta-analysis (due to the heterogenous weight loss outcome measurement) reported that the intervention group lost 1.8 kg (95% CI −4.3 to 0.8) more than the comparator group. Greater short-term effects are associated with the use of BCT 1.4 ‘action planning’ and the BCT 3.2 ‘social support (practical)’. The evidence is very uncertain also at long-term follow-ups (*k* = 2).
Health-related quality of life follow-up: mean 17 weeks	SMD **0.29 SD higher** (0.17 higher to 0.42 higher)	1052 (10 RCTs)	⨁⨁⨁◯ MODERATE^b^	Behavioural intervention likely increases the health-related quality of life slightly. At long-term follow-ups, the effect seems to diminish slightly (*k* = 2), but the evidence is uncertain.
Physical function follow-up: mean 12 weeks	SMD **0.42 SD higher** (0.12 higher to 0.73 higher)	1042 (10 RCTs)	⨁⨁◯◯ LOW^a,b^	Behavioural intervention likely increases physical function slightly. Increasing age, a higher proportion of male participants, and interventions using structured exercise sessions reported higher effect sizes at the end-treatment follow-ups. Interventions, including structured exercise sessions, reported a moderate and possibly clinically relevant improvement compared to interventions without structured exercise sessions. Using the BCT ‘Monitoring of outcome of behaviour without feedback’ and a higher number of BCT used for ‘Goals Settings and Planning’ was associated with lower effect sizes at the end-treatment follow-ups. At long-term follow-ups (*k* = 1), the effects seemed sustained.
Depression symptoms follow-up: mean 14 weeks	SMD **0.7 SD lower** (0.97 lower to 0.42 lower)	1038 (11 RCTs)	⨁⨁⨁◯ MODERATE^a^	Behavioural intervention likely reduces depression symptoms. Studies including people with a higher BMI, using a higher number of BCTs for ‘goal setting and planning’ and using the BCT ‘feedback and monitoring without feedback’, were associated with a lower reduction of depression symptoms. Depression severity was not associated with effect sizes. At the long-term follow-ups, the effect of behavioural intervention diminished.

*CI* confidence interval, *SMD* standardised mean difference, *MD* mean difference

GRADE Working Group grades of evidence

**High certainty**: We are very confident that the true effect lies close to that of the estimate of the effect

**Moderate certainty**: We are moderately confident in the effect estimate: The true effect is likely to be close to the estimate of the effect, but there is a possibility that it is substantially different

**Low certainty**: Our confidence in the effect estimate is limited: The true effect may be substantially different from the estimate of the effect

**Very low certainty**: We have very little confidence in the effect estimate: The true effect is likely to be substantially different from the estimate of effect

*Explanations*

^*^The risk in the intervention group (and its 95% confidence interval) is based on the assumed risk in the comparison group and the relative effect of the intervention (and its 95% CI)

^a^Quality of evidence downgraded of one level for inconsistency of the estimates

^b^Quality of evidence downgraded of one level for indirectness of the population

^c^Quality of evidence downgraded of one level for imprecision of the estimates

^d^Quality of the evidence downgraded of one level for inconsistency of the outcome measurements

## Discussion

This systematic review included 14 papers from 7 countries and a total of 1,378 people with multimorbidity. On average, behavioural interventions targeting lifestyle behaviours may improve health-related quality of life and physical function, reduce depression symptoms, and may have little to no effect on physical activity (although the 95% CI includes both important benefit and important harm), and weight loss in people with multimorbidity. However, the benefits diminish over time after the interventions ended, as shown by the long-term assessment meta-analyses.

### Overall Results in Context

The small improvements for physical activity and weight loss observed are comparable to the short- and long-term improvements seen in behavioural interventions including people with single chronic diseases such as osteoarthritis [51], diabetes [52], heart disease [53], depression, [54] and chronic obstructive pulmonary disease [55]. A possible explanation for these findings is the lack of adherence to the intervention after the studies end. However, greater short-term effects on physical activity and weight loss may be achieved by using the BCT ‘action planning’ and the BCT ‘social support (practical)’, which may potentially have an impact on long-term benefits as well [56]. Nevertheless, the few studies included and the nature of the exploratory analysis prevented us from upgrading the confidence we have in these results. The benefits of behavioural interventions on physical and psychosocial outcomes observed in this systematic review are greater than the findings from a previous systematic review focusing on interventions in multimorbidity in general [17]. The focus on specific combinations of conditions, in our systematic review, may partially explain the differences in results between the two systematic reviews. However, direct comparisons of these findings should be interpreted with caution due to the different populations of the two systematic reviews.

Studies using exercise therapy as part of the behavioural interventions appeared to promote clinically relevant improvements in physical function. This is in line with another systematic review focusing on exercise therapy in people with multimorbidity [15], which found a clinically relevant improvement in physical function following exercise therapy. Furthermore, studies including a higher proportion of males or older people and studies focusing on one BCT for ‘goals and planning’ relative to studies focusing on two or three BCTs for ‘goals and planning’ reported lower improvements in physical function. Similarly, using a higher number of BCTs for ‘goals and planning’ and ‘feedback and monitoring’ may reduce the effect of behavioural interventions on depression symptoms. This may be partially explained by the fact that focusing on many goals and being monitored in many (multiple/various) aspects may be too burdensome for some patients. This is in line with the results of a systematic review investigating the association between BCTs and adherence to exercise in patients with persistent musculoskeletal pain, which is an issue that is also common in people with multimorbidity [57]. Finally, a higher reduction of depression symptoms was seen in people with lower BMI. However, since very few studies were included, this limits our confidence in these results.

It is unclear why interventions targeting lifestyle behaviours, including physical activity and weight loss, improve physical and psychosocial outcomes (e.g., HRQoL and depression symptoms) but not necessarily behavioural outcomes. In this systematic review, two studies did not report an improvement in physical activity [38, 45]. A possible explanation may be that either light intensity activities or sedentary time were not captured as they reported only the time spent performing moderate to vigorous activity [45]. By contrast, increasing physical activity, although being a targeted behaviour of the intervention, was not the primary goal of the study [38]. Physical activity may improve in people with multimorbidity when the intervention explicitly focuses on improving it [58]. Additionally, another possible explanation is that patients may have improved their HRQoL or depression symotoms not necessarily by being more physically active or by losing weight but by adhering to one or more of the other targeted behaviours of the intervention such as quitting smoking, medication adherence, and/or engaging with others. Finally, in dealing with multiple morbidities, patients’ mental representations of their health are more complex. As proposed by the Common-Sense Model of Self-Regulation [59] which is a theoretical model that explicates the processes by which individuals respond to and manage a health threat, the model proposes that individuals navigate affective responses by formulating perceptions of the threat and potential treatment actions, creating action plans for addressing the threat, and integrating continuous feedback on action plan efficacy and threat-progression. People with multimorbidity likely to deal with both the health threat that their conditions present, but also how the threat makes them feel. Our results suggest that more emphasis is put on the latter to improve psychosocial outcomes, including depression symptoms, rather than directing attention to only reducing the threat by engaging in more physical activity.

### Research Implications

Behaviour change has been suggested to be contingent on both the capability, willingness, and readiness of the individual [60] and interventions that factor in all these, and recognise the equal status of intra-psychic and external factors in controlling behaviour may be more successful/effective. Therefore, when developing future interventions, a (socio)ecological theoretical approach that take this complexity into account by acknowledging an interplay between factors at the intrapersonal, interpersonal, organisational, community, and public policy levels should be applied [60]. Particularly, we suggest that future studies using behavioural interventions to improve physical activity should test the BCTs and clusters of BCTs that appear to be associated with greater improvements and focus on people with combinations of conditions linked by common risk factors and pathogenesis. Additionally, since the short-term benefits diminish over time, possibly due to lack of adherence to the interventions once the trial has ended. We suggest that future studies to focus on strategies that may help patients adhere to the effective interventions, as well as the investigation of interactions among BCTs, even after the intervention is finished (terminated/completed/discontinued). Similarly, attention should be paid to the mode of delivery of the intervention, which seems to play an important role in behavioural interventions [61–63]. Furthermore, the content of the interventions received by the comparator groups was often not reported in sufficient details. This is unfortunately common [64], and we suggest that authors of future studies follow, for example, the TIDieR checklist also for reporting comparator groups interventions [30]. Also, we suggest that future studies also measure changes of light intensity physical activity as well as sedentary time, in line with the 2020 WHO guidelines for physical activity [65], and include follow-up assessment close to 12 months and beyond to assess the effect of behavioural intervention over time. Yet, people with multimorbidity experience more health issues than people with single chronic diseases, and this includes physical, psychosocial, and cognitive problems [66]. Finally, given that the majority of the included RCTs included White participants of high SES, we suggest future studies to also focus on a wider range of ethnic populations across different SES [67]. This may change the confidence we have in these results, because people with different ethnic backgrounds and SES may respond differently to a behavioural intervention [68–71]. This should be considered when planning new interventions, and involving patients in the design of trials may help to improve feasibility and acceptability of the interventions.

### Clinical Implications

To improve physical activity in people with multimorbidity, health care professionals should consider encouraging, educating, and planning together with the patients on what physical activity to do, when, and how (BCT ‘action planning’). Further, health care professionals should advise or provide them with practical social support (BCT ‘social support (practical)’, e.g., provide a membership to a fitness centre and support by a qualified professional trained to deliver exercise therapy such as a physiotherapist or exercise physiologist). This may also help to achieve weight loss. To achieve greater improvements on physical function, we suggest focusing on one of the BCTs for ‘goals and planning’ rather than two or three. Also, it is advisable to avoid observing or recording outcomes of behaviour (e.g., physical activity) without providing feedback which appears to be associated with lower improvements in physical function. Similarly, using a higher number of BCTs for ‘goals and planning’ and ‘feedback and monitoring’ may reduce the effect of behavioural interventions on depression symptoms. Finally, particular attention should be paid to people with higher BMI, as they seem to be the subgroup of people with multimorbidity who benefit the least from reducing depression symptoms from behavioural interventions [72].

### Strengths and Limitations

The strengths of this systematic review are that we followed the Cochrane Handbook recommendations for performing it and the PRISMA guidelines for reporting it, contacted authors of the included studies to retrieve additional data about their studies, prespecified the main analyses, and followed a structured procedure to code BCTs. There are also limitations. Firstly, the scsarcity of studies matching our inclusion criteria is reflected in the inconsistency of the estimates of the meta-analyses and gave us low power for conducting the meta-regression analyses for physical activity and weight loss. However, we provided a narrative synthesis to investigate the associations between BCTs and these outcomes, thereby providing the readers with useful data applicable in clinical practice and research [32, 33, 73]. Secondly, among the studies reporting socioeconomic status and ethnicity included people of white ethnicity, with a high socio-economic status and with depression and heart failure, and very few studies with other common combination of conditions, limiting the generalizability of the findings to the entire multimorbid population [74, 75]. Finally, we potentially missed some of the BCTs used in the comparator groups who received usual care due to poor reporting of comparator interventions and due to not including digital health interventions, which however, is the focus of our current ongoing work [76].

## Conclusions

Behavioural interventions targeting lifestyle behaviours appear to have, on average, little or no effect on physical activity and weight loss in people with multimorbidity. By contrast, they improve health-related quality of life and physical function and reduce depression symptoms. Greater improvements in physical activity and weight loss are associated with using of the BCTs ‘action planning’ and ‘social support (practical)’. These benefits diminished after the interventions terminated, highlighting the importance of further studies investigating strategies to maintain behaviour change and long-term effects.

## Supplementary Information

Below is the link to the electronic supplementary material.

Supplementary file1 (XLSX 31 KB)

Supplementary file2 (DOCX 924 KB)
