# Supplementary material for: Effect of In-Person Delivered Behavioural Interventions in People with Multimorbidity: Systematic Review and Meta-analysis
Source: Int J Behav Med. 2022 Apr 28;30(2):167–89. doi: 10.1007/s12529-022-10092-8 (PMC10036283; doi:10.1007/s12529-022-10092-8)
Supplement: Supplementary file 2 — Supplementary file2 (DOCX 924 KB) [file 12529_2022_10092_MOESM2_ESM.docx]

**Supplementary material:**

**Effect of behavioural interventions in people with multimorbidity: systematic review and meta-analysis**

Table of Contents

[Additional File 1 -- Search strategies 2](#_Toc80286789)

[Additional File 2 PRISMA 2020 flow chart. 22](#_Toc80286790)

[Additional File 3. Table 1 (Behavior Change Techniques used in the individual studies) 24](#_Toc80286791)

[Additional File 4. BCT effectiveness ratio summary 26](#_Toc80286792)

[Additional File 4. Weight change meta-analysis at long term follow-up 27](#_Toc80286793)

[Additional File 5. Health-related quality of life meta-analysis at long term follow-up 27](#_Toc80286794)

[Additional File 6. Meta-regression participants, trials, interventions and BCT characteristics 28](#_Toc80286795)

[Additional File 7. Sub-group analysis on physical function 30](#_Toc80286796)

[Additional File 8. Risk of Bias summary 30](#_Toc80286797)

[Additional File 9. Small study bias. 31](#_Toc80286798)

[Additional File 10. STATA commands to reproduce the analyses 31](#_Toc80286799)

## Additional File 1 -- Search strategies

**MEDLINE vis PubMed**

1. osteoarthritis[MeSH Terms] OR osteoarthritis[Title/Abstract] OR osteoarthrit*[Title/Abstract] OR osteoarthros*[Title/Abstract] AND "Diabetes Mellitus"[Mesh] OR "Diabetes Mellitus, Type 2"[Mesh] OR Diabetes Mellitus[Title/Abstract] OR NIDDM[Title/Abstract] OR "Depression"[Mesh] OR "Dysthymic Disorder"[Mesh] OR Dysthymic Disorder[Title/Abstract] OR depress*[Title/Abstract] OR dysthymi* OR affect disorder[Title/Abstract] OR "Pulmonary Disease, Chronic Obstructive"[Mesh] OR "Pulmonary Emphysema"[Mesh] OR "Bronchitis, Chronic"[Mesh] OR COPD[Title/Abstract] OR Pulmonary Emphysema[Title/Abstract] OR Chronic Bronchitis[Title/Abstract] OR Chronic Obstructive Lung Disease[Title/Abstract] OR "Myocardial Ischemia"[Mesh] OR "Coronary Artery Disease"[Mesh] OR "Coronary Disease"[Mesh] OR "Myocardial Infarction"[Mesh] OR "Heart Failure"[Mesh] OR Myocardial Ischemia[Title/Abstract] OR Coronary Artery Disease[Title/Abstract] OR Myocardial Infarction[Title/Abstract] OR Heart Failure[Title/Abstract] OR Heart Diseases[Title/Abstract] OR "Hypertension"[Mesh] OR Hypertension[Title/Abstract] OR Hypertens*[Title/Abstract] OR High Blood Pressure[Title/Abstract] OR co-existing health problem*[Title/Abstract] OR co-existing illness[Title/Abstract] OR comorbid condition*[Title/Abstract] OR co-morbid condition[Title/Abstract] OR comorbid disease*[Title/Abstract] OR co-morbid disease[Title/Abstract] OR concurrent disease*[Title/Abstract] OR co-occurring condition*[Title/Abstract] OR cooccurring condition*[Title/Abstract] OR multiple diseases[Title/Abstract] OR comorbidity[Title/Abstract] OR comorbidities[Title/Abstract] OR multi-morbidity[Title/Abstract] OR multi-morbidities[Title/Abstract] OR polypathology[Title/Abstract] OR multidisease[Title/Abstract] OR multi-disease[Title/Abstract] OR "Comorbidity"[Mesh] OR "Multimorbidity"[Mesh] OR "Multiple Chronic Conditions"[Mesh] OR "Noncommunicable Diseases"[Mesh] OR "Diabetes Mellitus"[Mesh] OR "Diabetes Mellitus, Type 2"[Mesh] OR Diabetes Mellitus[Title/Abstract] OR NIDDM[Title/Abstract] AND "Depression"[Mesh] OR "Dysthymic Disorder"[Mesh] OR Dysthymic Disorder[Title/Abstract] OR depress*[Title/Abstract] OR dysthymi*OR affect disorder[Title/Abstract] OR "Pulmonary Disease, Chronic Obstructive"[Mesh] OR "Pulmonary Emphysema"[Mesh] OR "Bronchitis, Chronic"[Mesh] OR COPD[Title/Abstract] OR Pulmonary Emphysema[Title/Abstract] OR Chronic Bronchitis[Title/Abstract] OR Chronic Obstructive Lung Disease[Title/Abstract] OR "Myocardial Ischemia"[Mesh] OR "Coronary Artery Disease"[Mesh] OR "Coronary Disease"[Mesh] OR "Myocardial Infarction"[Mesh] OR "Heart Failure"[Mesh] OR Myocardial Ischemia[Title/Abstract] OR Coronary Artery Disease[Title/Abstract] OR Myocardial Infarction[Title/Abstract] OR Heart Failure[Title/Abstract] OR Heart Diseases[Title/Abstract] OR "Hypertension"[Mesh] OR Hypertension[Title/Abstract] OR Hypertens*[Title/Abstract] OR High Blood Pressure[Title/Abstract] OR co-existing health problem*[Title/Abstract] OR co-existing illness[Title/Abstract] OR comorbid condition*[Title/Abstract] OR co-morbid condition[Title/Abstract] OR comorbid disease*[Title/Abstract] OR co-morbid disease[Title/Abstract] OR concurrent disease*[Title/Abstract] OR co-occurring condition*[Title/Abstract] OR cooccurring condition*[Title/Abstract] OR multiple diseases[Title/Abstract] OR comorbidity[Title/Abstract] OR comorbidities[Title/Abstract] OR multi-morbidity[Title/Abstract] OR multi-morbidities[Title/Abstract] OR polypathology[Title/Abstract] OR multidisease[Title/Abstract] OR multi-disease[Title/Abstract] OR "Comorbidity"[Mesh] OR "Multimorbidity"[Mesh] OR "Multiple Chronic Conditions"[Mesh] OR "Noncommunicable Diseases"[Mesh] OR "Depression"[Mesh] OR "Dysthymic Disorder"[Mesh] OR Dysthymic Disorder[Title/Abstract] OR depress*[Title/Abstract] OR dysthymi*OR affect disorder[Title/Abstract] AND "Pulmonary Disease, Chronic Obstructive"[Mesh] OR "Pulmonary Emphysema"[Mesh] OR "Bronchitis, Chronic"[Mesh] OR COPD[Title/Abstract] OR Pulmonary Emphysema[Title/Abstract] OR Chronic Bronchitis[Title/Abstract] OR Chronic Obstructive Lung Disease[Title/Abstract] OR "Myocardial Ischemia"[Mesh] OR "Coronary Artery Disease"[Mesh] OR "Coronary Disease"[Mesh] OR "Myocardial Infarction"[Mesh] OR "Heart Failure"[Mesh] OR Myocardial Ischemia[Title/Abstract] OR Coronary Artery Disease[Title/Abstract] OR Myocardial Infarction[Title/Abstract] OR Heart Failure[Title/Abstract] OR Heart Diseases[Title/Abstract] OR "Hypertension"[Mesh] OR Hypertension[Title/Abstract] OR Hypertens*[Title/Abstract] OR High Blood Pressure[Title/Abstract] OR co-existing health problem*[Title/Abstract] OR co-existing illness[Title/Abstract] OR comorbid condition*[Title/Abstract] OR co-morbid condition[Title/Abstract] OR comorbid disease*[Title/Abstract] OR co-morbid disease[Title/Abstract] OR concurrent disease*[Title/Abstract] OR co-occurring condition*[Title/Abstract] OR cooccurring condition*[Title/Abstract] OR multiple diseases[Title/Abstract] OR comorbidity[Title/Abstract] OR comorbidities[Title/Abstract] OR multi-morbidity[Title/Abstract] OR multi-morbidities[Title/Abstract] OR polypathology[Title/Abstract] OR multidisease[Title/Abstract] OR multi-disease[Title/Abstract] OR "Comorbidity"[Mesh] OR "Multimorbidity"[Mesh] OR "Multiple Chronic Conditions"[Mesh] OR "Noncommunicable Diseases"[Mesh] OR "Pulmonary Disease, Chronic Obstructive"[Mesh] OR "Pulmonary Emphysema"[Mesh] OR "Bronchitis, Chronic"[Mesh] OR COPD[Title/Abstract] OR Pulmonary Emphysema[Title/Abstract] OR Chronic Bronchitis[Title/Abstract] OR Chronic Obstructive Lung Disease[Title/Abstract] AND "Myocardial Ischemia"[Mesh] OR "Coronary Artery Disease"[Mesh] OR "Coronary Disease"[Mesh] OR "Myocardial Infarction"[Mesh] OR "Heart Failure"[Mesh] OR Myocardial Ischemia[Title/Abstract] OR Coronary Artery Disease[Title/Abstract] OR Myocardial Infarction[Title/Abstract] OR Heart Failure[Title/Abstract] OR Heart Diseases[Title/Abstract] OR "Hypertension"[Mesh] OR Hypertension[Title/Abstract] OR Hypertens*[Title/Abstract] OR High Blood Pressure[Title/Abstract] OR co-existing health problem*[Title/Abstract] OR co-existing illness[Title/Abstract] OR comorbid condition*[Title/Abstract] OR co-morbid condition[Title/Abstract] OR comorbid disease*[Title/Abstract] OR co-morbid disease[Title/Abstract] OR concurrent disease*[Title/Abstract] OR co-occurring condition*[Title/Abstract] OR cooccurring condition*[Title/Abstract] OR multiple diseases[Title/Abstract] OR comorbidity[Title/Abstract] OR comorbidities[Title/Abstract] OR multi-morbidity[Title/Abstract] OR multi-morbidities[Title/Abstract] OR polypathology[Title/Abstract] OR multidisease[Title/Abstract] OR multi-disease[Title/Abstract] OR "Comorbidity"[Mesh] OR "Multimorbidity"[Mesh] OR "Multiple Chronic Conditions"[Mesh] OR "Noncommunicable Diseases"[Mesh] OR "Myocardial Ischemia"[Mesh] OR "Coronary Artery Disease"[Mesh] OR "Coronary Disease"[Mesh] OR "Myocardial Infarction"[Mesh] OR "Heart Failure"[Mesh] OR Myocardial Ischemia[Title/Abstract] OR Coronary Artery Disease[Title/Abstract] OR Myocardial Infarction[Title/Abstract] OR Heart Failure[Title/Abstract] OR Heart Diseases[Title/Abstract] AND "Hypertension"[Mesh] OR Hypertension[Title/Abstract] OR Hypertens*[Title/Abstract] OR High Blood Pressure[Title/Abstract] OR co-existing health problem*[Title/Abstract] OR co-existing illness[Title/Abstract] OR comorbid condition*[Title/Abstract] OR co-morbid condition[Title/Abstract] OR comorbid disease*[Title/Abstract] OR co-morbid disease[Title/Abstract] OR concurrent disease*[Title/Abstract] OR co-occurring condition*[Title/Abstract] OR cooccurring condition*[Title/Abstract] OR multiple diseases[Title/Abstract] OR comorbidity[Title/Abstract] OR comorbidities[Title/Abstract] OR multi-morbidity[Title/Abstract] OR multi-morbidities[Title/Abstract] OR polypathology[Title/Abstract] OR multidisease[Title/Abstract] OR multi-disease[Title/Abstract] OR "Comorbidity"[Mesh] OR "Multimorbidity"[Mesh] OR "Multiple Chronic Conditions"[Mesh] OR "Noncommunicable Diseases"[Mesh]
2. "Health Behavior"[Mesh] OR "Patient Education as Topic"[Mesh] OR "Self Care"[Mesh] OR "Self-Management"[Mesh] OR "Disease Management"[Mesh] OR "Health Promotion"[Mesh] OR "Social Support"[Mesh] OR "Behavior Therapy"[Mesh] OR "Self Efficacy"[Mesh] OR "Motivation"[Mesh] OR "Primary Prevention"[Mesh] OR behaviour* AND change[Title/Abstract] OR behaviour* AND technique[Title/Abstract] OR behaviour* AND intervention[Title/Abstract] OR behaviour* AND health[Title/Abstract] OR education* AND program[Title/Abstract] OR education* AND information[Title/Abstract] OR education* AND health[Title/Abstract] OR health promotion[Title/Abstract] OR coping[Title/Abstract] OR social support[Title/Abstract] OR self management[Title/Abstract] OR motivation[Title/Abstract] OR self-efficacy[Title/Abstract]
3. Exercise[MeSH Terms] OR Sports[MeSH Terms] OR Sports[Title/Abstract] OR Work-out[Title/Abstract] OR Workout[Title/Abstract] OR Working out[Title/Abstract] OR Aerobics[Title/Abstract] OR Physical Fitness[MeSH Terms] OR Fitness[Title/Abstract] OR Physical conditioning[Title/Abstract] OR Physically fit[Title/Abstract] OR Active lifestyle[Title/Abstract] OR Physical activity[Title/Abstract] OR Exercise[Title/Abstract] OR Exercise movement techniques[MeSH Terms] OR Exercise movement techniques[Title/Abstract] OR Exercise therapy[MeSH Terms] OR Exercise therapy[Title/Abstract] OR Locomotion[MeSH Terms] OR Locomotion[Title/Abstract] OR Physical Therapy Modalities[MeSH Terms] OR Physical Therapy Modalities[Title/Abstract] OR Physiotherapy[Title/Abstract] OR Physical therapy[Title/Abstract] OR Physical Education and Training[MeSH Terms] OR Physical Education[Title/Abstract] AND Training[Title/Abstract] OR Motor Activity[MeSH Terms] OR Motor Activity[Title/Abstract] OR Resistance Training[Title/Abstract] OR Strengthening[Title/Abstract] OR Muscle strength[MeSH Terms] OR Muscle strength[Title/Abstract] OR Walking[Title/Abstract] OR Running[Title/Abstract] OR Jogging[Title/Abstract] OR Cycling[Title/Abstract] OR Bicycling[Title/Abstract] OR Swimming[Title/Abstract] OR Gymnastics[Title/Abstract] OR Yoga[Title/Abstract] OR Dancing[MeSH Terms] OR Dancing[Title/Abstract] OR Pilates[Title/Abstract] OR Body Mass Index[MeSH Terms] OR Body Mass Index[Title/Abstract] OR Body weight[MeSH Terms] OR Body weight[Title/Abstract] OR Weight loss[Title/Abstract] OR Overweight[Title/Abstract] OR Obesity[Title/Abstract] OR Health promotion[MeSH Terms] OR Health promotion[Title/Abstract] OR Adiposity[Title/Abstract] OR Adiposity[MeSH Terms] OR Excess body fat[Title/Abstract] OR Activities of daily living[MeSH Terms] OR Activities of daily living[Title/Abstract] OR ADL[Title/Abstract] OR functional ability[Title/Abstract] OR physical limitations[Title/Abstract] OR physical function[Title/Abstract] OR functional assessment[Title/Abstract] OR Health status[MeSH Terms] OR Health status[Title/Abstract] OR Quality of life[Title/Abstract] OR HRQOL[Title/Abstract]
4. randomized controlled trial[pt] OR controlled clinical trial[pt] OR randomized[tiab] OR placebo[tiab] OR drug therapy[sh] OR randomly[tiab] OR trial[tiab] OR groups[tiab] NOT animals[mh] NOT humans[mh]
5. 1 and 2 and 3 and 4
6. Filters: from 2000 – 2020

**EMBASE (OVID)**

1. Multimorbidity/
2. Multimorbid*.ti,ab.
3. Multi-morbid*.ti,ab.
4. Multidisease?.ti,ab.
5. Multi-disease?.ti,ab.
6. Multiple disease?.ti,ab.
7. Multiple diagno*.ti,ab.
8. Multicondition?.ti,ab.
9. ((Multi or multiple) adj2 (morbid* or disease? or condition? or ill*OR syndrom* or symptom* or disorder? or health)).ti,ab.
10. ((Associated or Co-existing or Concurrent or Cooccurring or Chronic*) adj (morbid* or disease? or condition? or ill*OR syndrom* or symptom* or disorder? or health)).ti,ab.
11. Polypathology.ti,ab.
12. pluripathology.ti,ab.
13. Comorbidity/
14. Comorbid*.ti,ab.
15. (Pattern* adj2 disease?).ti,ab.
16. (Cluster* adj2 disease?).ti,ab.
17. Co-morbid*.ti,ab.
18. Multiple Chronic Conditions/
19. Noncommunicable Diseases/
20. 1 or 2 or 3 or 4 or 5 or 6 or 7 or 8 or 9 or 10 or 11 or 12 or 13 or 14 or 15 or 16 or 17 or 18 or 19
21. Hypertension/
22. hypertension.ti,ab.
23. hypertens*.ti,ab.
24. high blood pressure.ti,ab.
25. 21 or 22 or 23 or 24
26. myocardial ischemia/ or acute coronary syndrome/ or coronary disease/ or myocardial infarction/
27. Coronary Artery Disease/
28. (myocard* adj (ischem* or ischaem* or infact*)).ti,ab.
29. ((ischem* or ischaem*) adj heart disease).ti,ab.
30. Heart Failure/
31. Heart Failure.ti,ab.
32. 26 or 27 or 28 or 29 or 30 or 31
33. exp Pulmonary Disease, Chronic Obstructive/
34. COPD.ti,ab.
35. Chronic Obstructive Lung Disease.ti,ab.
36. Chronic bronchitis.ti,ab.
37. Pulmonary emphysema.ti,ab.
38. 33 or 34 or 35 or 36 or 37
39. exp Depression/
40. Depress*.ti,ab.
41. depressive disorder, major/ or dysthymic disorder/
42. dysthymic disorder.ti,ab.
43. dysthymi*.ti,ab.
44. affect* disorder.ti,ab.
45. 39 or 40 or 41 or 42 or 43 or 44
46. diabetes mellitus/ or diabetes mellitus, type 2/
47. Type 2 diab*.ti,ab.
48. Type II diab*.ti,ab.
49. Non-Insulin-Dependent Diabetes Mellitus.ti,ab.
50. NIDDM.ti,ab.
51. diabetes mellitus.ti,ab.
52. 46 or 47 or 48 or 49 or 50 or 51
53. exp Osteoarthritis/
54. Osteoarthritis.ti,ab.
55. Osteoarthr$.ti,ab.
56. (degenerative adj2 arthritis).ti,ab.
57. arthrosis.ti,ab.
58. 53 or 54 or 55 or 56 or 57
59. Patient Education as Topic/
60. exp Health Education/
61. ((health or patient$) adj2 (educat$ or information)).ti,ab.
62. exp Self Care/
63. ((self or symptom$) adj (care or help or manag$ or directed or monitor$ or efficacy or admin$)).ti,ab.
64. exp Health Behavior/
65. exp Life Style/
66. (Behav$ adj (health* or intervention? or chang* or modification? or modify* or therap* or program* or technique*)).ti,ab.
67. (Life style adj (intervention? or chang* or modification? or modify* or therap* or program* or technique*)).ti,ab.
68. exp Behavior Therapy/
69. (Educat$ adj (program* or intervention* or meet* or session? or workshop? or material? or method? or information or program)).ti,ab.
70. Self Management/
71. Self Manage$.ti,ab.
72. Social support/
73. (Social adj (support or network$)).ti,ab.
74. Motivation/ or Motivation*.ti,ab.
75. Secondary prevention/ or Secondary prevention.ti,ab.
76. (Cope or copes).mp. or coping.ti,ab.
77. exp self efficacy/
78. 59 or 60 or 61 or 62 or 63 or 64 or 65 or 66 or 67 or 68 or 69 or 70 or 71 or 72 or 73 or 74 or 75 or 76 or 77
79. randomized controlled trial.pt.
80. controlled clinical trial.pt.
81. randomized.ab.
82. placebo.ab.
83. drug therapy.fs.
84. randomly.ab.
85. trial.ab.
86. groups.ab.
87. 79 or 80 or 81 or 82 or 83 or 84 or 85 or 86
88. exp animals/ not humans.sh.
89. 25 and (20 or 32 or 38 or 45 or 52 or 58)
90. 32 and (20 or 38 or 45 or 52 or 58)
91. 38 and (20 or 45 or 52 or 58)
92. 45 and (20 or 52 or 58)
93. 52 and (20 or 58)
94. 89 or 90 or 91 or 92 or 93
95. 78 and 87 and 94
96. exp physical activity/
97. Physical activity.ti,ab.
98. Physical activ*.ti,ab.
99. exp Sports/
100. Sport*.ti,ab.
101. Work-out.ti,ab.
102. Workout.ti,ab.
103. Working out.ti,ab.
104. Aerobics.ti,ab.
105. exp conditioning/ or exp exercise/ or fitness/
106. conditioning.ti,ab.
107. exercise.ti,ab.
108. Exercis*.ti,ab.
109. fitness.ti,ab.
110. exp locomotion/
111. locomotion.ti,ab.
112. exp physiotherapy/
113. Physiotherapy.ti,ab.
114. exp motor activity/
115. Motor Activity.ti,ab.
116. exp training/ or exp resistance training/ or exp muscle strength/
117. training.ti,ab.
118. resistance training.ti,ab.
119. muscle strength.ti,ab.
120. exp walking/
121. walking.ti,ab.
122. running.ti,ab.
123. exp jogging/
124. Jogging.ti,ab.
125. exp cycling/
126. Cycling.ti,ab.
127. exp running/
128. exp swimming/
129. Swimming.ti,ab.
130. exp physical education/
131. physical education.ti,ab.
132. exp yoga/
133. yoga.ti,ab.
134. exp dancing/
135. Dancing.ti,ab.
136. exp pilates/
137. pilates.ti,ab.
138. exp body mass/
139. Body Mass Index.ti,ab.
140. exp body weight/
141. Body weight.ti,ab.
142. exp body weight loss/
143. body weight loss.ti,ab.
144. exp obesity/
145. obesity.ti,ab.
146. obes*.ti,ab.
147. exp health promotion/
148. Health promotion.ti,ab.
149. exp daily life activity/
150. daily life activity.ti,ab.
151. exp ADL disability/
152. ADL disability.ti,ab.
153. exp functional status/
154. functional status.ti,ab.
155. exp physical disability/
156. physical disability.ti,ab.
157. exp physical performance/
158. physical performance.ti,ab.
159. exp functional assessment/
160. functional assessment.ti,ab.
161. exp health status/
162. health status.ti,ab.
163. exp "quality of life"/
164. "quality of life".ti,ab.
165. 96 or 97 or 98 or 99 or 100 or 101 or 102 or 103 or 104 or 105 or 106 or 107 or 108 or 109 or 110 or 111 or 112 or 113 or 114 or 115 or 116 or 117 or 118 or 119 or 120 or 121 or 122 or 123 or 124 or 125 or 126 or 127 or 128 or 129 or 130 or 131 or 132 or 133 or 134 or 135 or 136 or 137 or 138 or 139 or 140 or 141 or 142 or 143 or 144 or 145 or 146 or 147 or 148 or 149 or 150 or 151 or 152 or 153 or 154 or 155 or 156 or 157 or 158 or 159 or 160 or 161 or 162 or 163 or 164
166. 95 and 165
167. limit 166 to (human and randomized controlled trial and yr="2019 - 2020")

**CINAHL (Ebsco)**

1. (TX “Multimorbidity”)
2. (TI "Multimorbid*") or (AB "Multimorbid*")
3. (TI "Multi-morbid*") or (AB "Multi-morbid*")
4. (TI "Multidisease?") or (AB "Multidisease?")
5. (TI "Multicondition?") or (AB "Multicondition?")
6. ((Multi or multiple) N2 (morbid* or disease? or condition? or ill* or syndrom* or disorder?))
7. (TX "Multiple Chronic Conditions")
8. (MM "Comorbidity")
9. (TI "Comorbid*") OR (AB "Comorbid*")
10. (TI "Co-morbid*") OR (AB "Co-morbid*")
11. (MM "Chronic Disease+")
12. ((Chronic*) N0 (disease? or ill* or care or condition? or disorder? or health or medication* or syndrom* or symptom*))
13. S1 OR S2 OR S3 OR S4 OR S5 OR S6 OR S7 OR S8 OR S9 OR S10 OR S11 OR S12
14. (MH "Hypertension+")
15. (MH "Hypertension, Isolated Systolic")
16. (TI "high blood pressure") OR (AB "high blood pressure")
17. S14 OR S15 OR S16
18. (MH "Coronary Arteriosclerosis")
19. (MH "Heart Failure+")
20. (MH "Myocardial Infarction+")
21. S18 OR S19 OR S20
22. (MH "Pulmonary Disease, Chronic Obstructive+")
23. (TI "COPD") OR (AB "COPD")
24. S22 OR S23
25. (MH "Depression+")
26. (TI "major depressive disorder") OR (AB "major depressive disorder")
27. (TI "Chronic depression") OR (AB "Chronic depression")
28. S25 OR S26 OR S27
29. (MH "Diabetes Mellitus+")
30. (TI "Diabetes mellitus type 2") OR (AB "Diabetes mellitus type 2")
31. (TI "Non-Insulin-Dependent Diabetes Mellitus") OR (AB "Non-Insulin-Dependent Diabetes Mellitus")
32. (MH "Blood Glucose")
33. S29 OR S30 OR S31 OR S32
34. (MH “Osteoarthritis+”)
35. (MH "Osteoarthritis, Hip")
36. (MH "Osteoarthritis, Knee")
37. S34 OR S35 OR S36
38. (MM "Patient Education+")
39. ((patient$) N2 (educat$))
40. (MH "Health Education+")
41. (MH "Self Care+")
42. (MH "Health Behavior+")
43. (Behav$ N0 (health* or intervention? or chang* or modification? or modify* or therap* or program* or technique*))
44. (MH "Life Style+")
45. (Life style N0 (intervention? or chang* or modification? or modify* or therap* or program* or technique*))
46. (MH "Self-Management")
47. (MH "Support, Psychosocial+")
48. (Social) N0 (support or network$)
49. (MH "Motivation+") OR (TI "Motivation$") OR (AB "Motivation$")
50. (MH "Self-Efficacy")
51. S38 OR S39 OR S40 OR S41 OR S42 OR S43 OR S44 OR S45 OR S46 OR S47 OR S48 OR S49 OR S50
52. MH randomized controlled trials
53. MH double-blind studies
54. MH single-blind studies
55. MH random assignment
56. MH pretest-posttest design
57. MH cluster sample
58. TI (randomised OR randomized)
59. AB (random*)
60. TI (trial)
61. MH (sample size) AND AB (assigned OR allocated OR control)
62. MH (placebos)
63. PT (randomized controlled trial)
64. AB (control W5 group)
65. MH (crossover design) OR MH(comparative studies)
66. AB (cluster W3 RCT)
67. MH animals+
68. MH (animal studies)
69. TI (animal model*)
70. S67 OR S68 OR S69
71. MH (human)
72. S70 NOT S71
73. S52 OR S53 OR S54 OR S55 OR S56 OR S57 OR S58 OR S59 OR S60 OR S61 OR S62 OR S63 OR S64 OR S65 OR S66
74. S73 NOT S72
75. 13 AND (17 OR 21 OR 24 OR 28 OR 33 OR 37)
76. 17 AND (13 OR 21 OR 24 OR 28 OR 33 OR 37)
77. 21 AND (13 OR 17 OR 24 OR 28 OR 33 OR 37)
78. 24 AND (13 OR 17 OR 21 OR 28 OR 33 OR 37)
79. 28 AND (13 OR 17 OR 21 OR 24 OR 33 OR 37)
80. 33 AND (13 OR 17 OR 21 OR 24 OR 28 OR 33)
81. S75 OR S76 OR S77 OR S78 OR S79 OR S80
82. S81 AND S51 AND S74
83. MH physical activity
84. (TI "physical activity") or (AB "physical activity")
85. (TI "Physical activ*") or (AB "Physical activ*")
86. MH sports
87. (TI "Sports") or (AB "Sports")
88. (TI "Sport*") or (AB "Sport*")
89. (TI "Work-out") or (AB "Work-out")
90. (TI "Workout") or (AB "Workout")
91. (TI "Working out") or (AB "Working out")
92. (TI "Aerobics") or (AB "Aerobics")
93. MH physical fitness
94. (TI "physical fitness") or (AB "physical fitness")
95. MH exercise
96. (TI "exercise") or (AB "exercise")
97. (TI "exercis*") or (AB "exercis*")
98. MH locomotion
99. (TI "locomotion") or (AB "locomotion")
100. MH Physical Therapy
101. (TI "Physical Therapy") or (AB "Physical Therapy")
102. MH motor activity
103. (TI "motor activity") or (AB "motor activity")
104. MH resistance training
105. (TI "resistance training") or (AB "resistance training")
106. MH muscle strength
107. (TI "muscle strength") or (AB "muscle strength")
108. MH walking
109. (TI "walking") or (AB "walking")
110. MH Running
111. (TI "Running") or (AB "Running")
112. MH Jogging
113. (TI "Jogging") or (AB "Jogging")
114. MH Cycling
115. (TI "Cycling") or (AB "Cycling")
116. (TI "swimming") or (AB "swimming")
117. MH Gymnastics
118. (TI "Gymnastics") or (AB "Gymnastics")
119. (TI "Gymnastic*") or (AB "Gymnastic*")
120. MH Yoga
121. (TI "Yoga") or (AB "Yoga")
122. MH dancing
123. (TI "dancing") or (AB "dancing")
124. MH pilates
125. (TI "pilates") or (AB "pilates")
126. MH body mass index or bmi
127. (TI "body mass index") or (AB "body mass index") or (TI "bmi") or (AB "bmi")
128. MH body weight
129. (TI "body weight") or (AB "body weight")
130. MH obesity
131. (TI "obesity") or (AB "obesity")
132. (TI "obes*") or (AB "obes*")
133. MH health promotion
134. (TI "health promotion") or (AB "health promotion")
135. MH functional status
136. (TI "functional status") or (AB "functional status")
137. MH physical performance
138. (TI "physical performance") or (AB "physical performance")
139. MH functional assessment
140. (TI "functional assessment") or (AB "functional assessment")
141. MH health status
142. (TI "health status") or (AB "health status")
143. MH quality of life or well being or well-being or health-related quality of life
144. (TI "quality of life") or (AB "quality of life") or (TI "well being") or (AB "well being") or (TI "well-being") or (AB "well-being") or (TI "health-related quality of life") or (AB "health-related quality of life")
145. S83 OR S84 OR S85 OR S86 OR S87 OR S88 OR S89 OR S90 OR S91 OR S92 OR S93 OR S94 OR S95 OR S96 OR S97 OR S98 OR S99 OR S100 OR S101 OR S102 OR S103 OR S104 OR S105 OR S106 OR S107 OR S108 OR S109 OR S110 OR S111 OR S112 OR S113 OR S114 OR S115 OR S116 OR S117 OR S118 OR S119 OR S120 OR S121 OR S122 OR S123 OR S124 OR S125 OR S126 OR S127 OR S128 OR S129 OR S130 OR S131 OR S132 OR S133 OR S134 OR S135 OR S136 OR S137 OR S138 OR S139 OR S140 OR S141 OR S142 OR S143 OR S144
146. S82 AND S145
147. Filters: 2019-2020

**CENTRAL**

ID Search Hits

#1 MeSH descriptor: [Osteoarthritis] explode all trees 7557

#2 (Osteoarthritis):ti,ab,kw 18008

#3 (Osteoarthrit*):ti,ab,kw 18090

#4 (Osteoarthros*):ti,ab,kw 485

#5 #1 OR #2 OR #3 OR #4 18250

#6 (co-existing health problem*):ti,ab,kw 22

#7 (coexisting illness*):ti,ab,kw 93

#8 (co-existing illness*):ti,ab,kw 26

#9 (coexisting patholog*):ti,ab,kw 93

#10 (co-existing patholog*):ti,ab,kw 24

#11 (comorbid condition*):ti,ab,kw 2536

#12 (co-morbid condition*):ti,ab,kw 576

#13 (comorbid diagnos*):ti,ab,kw 2242

#14 (co-morbid diagnos*):ti,ab,kw 393

#15 (comorbid disease*):ti,ab,kw 2464

#16 (co-morbid disease*):ti,ab,kw 501

#17 (comorbid illness*):ti,ab,kw 894

#18 (concurrent disease*):ti,ab,kw 5041

#19 (co-occurring condition*):ti,ab,kw 376

#20 (cooccurring condition*):ti,ab,kw 379

#21 ("multiple conditions"):ti,ab,kw 53

#22 ("multiple disease"):ti,ab,kw 59

#23 ("multiple diseases"):ti,ab,kw 62

#24 MeSH descriptor: [Comorbidity] explode all trees 3573

#25 (Comorbidity):ti,ab,kw 13768

#26 ("comorbidities"):ti,ab,kw 8739

#27 (multimorbidity):ti,ab,kw 368

#28 MeSH descriptor: [Multimorbidity] explode all trees 43

#29 (multi-morbidity):ti,ab,kw 59

#30 (multi-morbidities):ti,ab,kw 8

#31 #6 OR #7 OR #8 OR #9 OR #10 OR #11 OR #12 OR #13 OR #14 OR #15 OR #16 OR #17 OR #18 OR #19 OR #20 OR #21 OR #22 OR #23 OR #24 OR #25 OR #26 OR #27 OR #28 OR #29 OR #30 27618

#32 MeSH descriptor: [Diabetes Mellitus] explode all trees 31544

#33 (diabetes mellitus):ti,ab,kw 64062

#34 MeSH descriptor: [Diabetes Mellitus, Type 2] explode all trees 17804

#35 (NIDDM):ti,ab,kw 1078

#36 (impaired glucose toleranc*):ti,ab,kw 3404

#37 MeSH descriptor: [Glucose Intolerance] explode all trees 1131

#38 MeSH descriptor: [Blood Glucose] explode all trees 16056

#39 #32 OR #33 OR #34 OR #35 OR #36 OR #37 OR #38 76808

#40 MeSH descriptor: [Depression] explode all trees 12317

#41 (depression):ti,ab,kw 76593

#42 MeSH descriptor: [Dysthymic Disorder] explode all trees 178

#43 (Dysthymic Disorder):ti,ab,kw 341

#44 (depress*):ti,ab,kw 86875

#45 (dysthymi*):ti,ab,kw 963

#46 (affect disorder*):ti,ab,kw 7239

#47 (affective symptom*):ti,ab,kw 2626

#48 #40 OR #41 OR #42 OR #43 OR #44 OR #45 OR #46 OR #47 93057

#49 MeSH descriptor: [Hypertension] explode all trees 18032

#50 (Hypertension):ti,ab,kw 60572

#51 (Hypertens*):ti,ab,kw 64852

#52 (High Blood Pressure):ti,ab,kw 23383

#53 MeSH descriptor: [Blood Pressure] explode all trees 27494

#54 #49 OR #50 OR #51 OR #52 OR #53 94871

#55 MeSH descriptor: [Pulmonary Disease, Chronic Obstructive] explode all trees 5771

#56 MeSH descriptor: [Pulmonary Disease, Chronic Obstructive] explode all trees 5771

#57 (COPD):ti,ab,kw 16418

#58 MeSH descriptor: [Pulmonary Emphysema] explode all trees 299

#59 (Pulmonary Emphysema):ti,ab,kw 936

#60 MeSH descriptor: [Pulmonary Disease, Chronic Obstructive] explode all trees 5771

#61 (COAD):ti,ab,kw 81

#62 MeSH descriptor: [Bronchitis, Chronic] explode all trees 170

#63 (Chronic Bronchitis):ti,ab,kw 2449

#64 (Chronic Obstructive Lung Disease):ti,ab,kw 10897

#65 #55 OR #56 OR #57 OR #58 OR #59 OR #60 OR #61 OR #62 OR #63 OR #64 21583

#66 MeSH descriptor: [Myocardial Ischemia] explode all trees 28424

#67 (Myocardial Ischemia):ti,ab,kw 10154

#68 MeSH descriptor: [Coronary Artery Disease] explode all trees 6518

#69 (Coronary Artery Disease):ti,ab,kw 22734

#70 MeSH descriptor: [Coronary Disease] explode all trees 13847

#71 (Coronary Disease):ti,ab,kw 34682

#72 MeSH descriptor: [Myocardial Infarction] explode all trees 11163

#73 (myocardial infarction):ti,ab,kw 30992

#74 MeSH descriptor: [Angina Pectoris] explode all trees 4576

#75 (Angina Pectoris):ti,ab,kw 9489

#76 MeSH descriptor: [Heart Failure] explode all trees 9474

#77 (Heart Failure):ti,ab,kw 36335

#78 (HFNEF):ti,ab,kw 18

#79 (HFPEF):ti,ab,kw 702

#80 (HFREF):ti,ab,kw 674

#81 ("HF PEF"):ti,ab,kw 31

#82 MeSH descriptor: [Heart Diseases] explode all trees 52679

#83 (Heart Diseases):ti,ab,kw 13580

#84 MeSH descriptor: [Coronary Artery Bypass] explode all trees 5442

#85 (Coronary Artery Bypass):ti,ab,kw 12586

#86 #66 OR #67 OR #68 OR #69 OR #70 OR #71 OR #72 OR #73 OR #74 OR #75 OR #76 OR #77 OR #78 OR #79 OR #80 OR #81 OR #82 OR #83 OR #84 OR #85 113642

#87 #5 AND (#31 OR #39 OR #48 OR #54 OR #65 OR #86) 1551

#88 #39 AND (#31 OR #5 OR #48 OR #54 OR #65 OR #86) 23119

#89 #48 AND (#31 OR #5 OR #39 OR #54 OR #65 OR #86) 16710

#90 #54 AND (#31 OR #5 OR #39 OR #48 OR #65 OR #86) 32504

#91 #65 AND (#31 OR #5 OR #39 OR #48 OR #54 OR #86) 3852

#92 #86 AND (#31 OR #5 OR #39 OR #48 OR #54 OR #65) 32201

#93 (#87 OR #88 OR #89 OR #90 OR #91 OR #92) 56010

#94 MeSH descriptor: [Health Behavior] explode all trees 35691

#95 MeSH descriptor: [Patient Education as Topic] explode all trees 8915

#96 MeSH descriptor: [Self Care] explode all trees 5649

#97 MeSH descriptor: [Self-Management] explode all trees 391

#98 MeSH descriptor: [Disease Management] explode all trees 4646

#99 MeSH descriptor: [Health Promotion] explode all trees 6574

#100 MeSH descriptor: [Social Support] explode all trees 3338

#101 MeSH descriptor: [Behavior Therapy] explode all trees 16685

#102 MeSH descriptor: [Self Efficacy] explode all trees 3138

#103 MeSH descriptor: [Motivation] explode all trees 8244

#104 MeSH descriptor: [Primary Prevention] explode all trees 4164

#105 (behaviour* AND change):ti,ab,kw 6763

#106 (behaviour* AND technique):ti,ab,kw 671

#107 (behaviour* AND intervention):ti,ab,kw 16137

#108 (behaviour* AND health):ti,ab,kw 12678

#109 (education* AND program):ti,ab,kw 27823

#110 (education* AND information):ti,ab,kw 13714

#111 (education* AND health):ti,ab,kw 41384

#112 (health promotion):ti,ab,kw 11446

#113 (coping):ti,ab,kw 8768

#114 (social support):ti,ab,kw 14503

#115 (self management):ti,ab,kw 19228

#116 (motivation):ti,ab,kw 12448

#117 (self-efficacy):ti,ab,kw 13060

#118 (#94 OR #95 OR #96 OR #97 OR #98 OR #99 OR #100 OR #101 OR #102 OR #103 OR #104 OR #105 OR #106 OR #107 OR #108 OR #109 OR #110 OR #111 OR #112 OR #113 OR #114 OR #115 OR #116 OR #117) 158392

#119 #93 AND #118 8290

#120 MeSH descriptor: [Exercise] explode all trees 24606

#121 MeSH descriptor: [Sports] explode all trees 15588

#122 (Sports):ti,ab,kw 6482

#123 (Work-out):ti,ab,kw 110

#124 (Workout):ti,ab,kw 463

#125 (Working out):ti,ab,kw 2255

#126 (Aerobics):ti,ab,kw 274

#127 MeSH descriptor: [Physical Fitness] explode all trees 3292

#128 (Fitness):ti,ab,kw 11206

#129 (Physical conditioning):ti,ab,kw 1319

#130 (Physically fit):ti,ab,kw 242

#131 (Active lifestyle):ti,ab,kw 2086

#132 (Physical activity):ti,ab,kw 41299

#133 (Exercise):ti,ab,kw 96684

#134 MeSH descriptor: [Exercise Movement Techniques] explode all trees 2124

#135 (Exercise movement techniques):ti,ab,kw 558

#136 MeSH descriptor: [Exercise Therapy] explode all trees 13799

#137 (Exercise therapy):ti,ab,kw 40256

#138 MeSH descriptor: [Locomotion] explode all trees 8178

#139 (Locomotion):ti,ab,kw 1052

#140 MeSH descriptor: [Physical Therapy Modalities] explode all trees 25626

#141 (Physical Therapy Modalities):ti,ab,kw 4532

#142 (Physiotherapy):ti,ab,kw 12275

#143 (Physical therapy):ti,ab,kw 47938

#144 MeSH descriptor: [Physical Education and Training] explode all trees 1599

#145 (Physical Education AND Training):ti,ab,kw 4886

#146 MeSH descriptor: [Motor Activity] explode all trees 27682

#147 (Motor Activity):ti,ab,kw 10172

#148 ("resistance training"):ti,ab,kw 9378

#149 (Strengthening):ti,ab,kw 5143

#150 MeSH descriptor: [Muscle Strength] explode all trees 5624

#151 (Muscle strength):ti,ab,kw 19628

#152 (Walking):ti,ab,kw 21973

#153 (Running):ti,ab,kw 6441

#154 (Jogging):ti,ab,kw 489

#155 (Cycling):ti,ab,kw 5553

#156 (Bicycling):ti,ab,kw 1735

#157 (Swimming):ti,ab,kw 1067

#158 (Gymnastics):ti,ab,kw 289

#159 (Yoga):ti,ab,kw 3407

#160 (Dancing):ti,ab,kw 535

#161 MeSH descriptor: [Dancing] explode all trees 172

#162 (Pilates):ti,ab,kw 654

#163 MeSH descriptor: [Body Mass Index] explode all trees 10136

#164 (Body Mass Index):ti,ab,kw 41376

#165 MeSH descriptor: [Body Weight] explode all trees 28140

#166 (Body weight):ti,ab,kw 63509

#167 (Weight loss):ti,ab,kw 25316

#168 (Overweight):ti,ab,kw 16769

#169 (Obesity):ti,ab,kw 36983

#170 MeSH descriptor: [Health Promotion] explode all trees 6574

#171 (Health promotion):ti,ab,kw 11446

#172 (Adiposity):ti,ab,kw 2650

#173 MeSH descriptor: [Adiposity] explode all trees 739

#174 (Excess body fat):ti,ab,kw 567

#175 MeSH descriptor: [Activities of Daily Living] explode all trees 9358

#176 (Activities of daily living):ti,ab,kw 11351

#177 (ADL):ti,ab,kw 3416

#178 (functional ability):ti,ab,kw 6264

#179 (physical limitations):ti,ab,kw 2985

#180 (physical function):ti,ab,kw 25584

#181 (functional assessment):ti,ab,kw 23540

#182 MeSH descriptor: [Health Status] explode all trees 29740

#183 (Health status):ti,ab,kw 37919

#184 (Quality of life):ti,ab,kw 117876

#185 (HRQOL):ti,ab,kw 5459

#186 (#120 OR #121 OR #122 OR #123 OR #124 OR #125 OR #126 OR #127 OR #128 OR #129 OR #130 OR #131 OR #132 OR #133 OR #134 OR #135 OR #136 OR #137 OR #138 OR #139 OR #140 OR #141 OR #142 OR #143 OR #144 OR #145 OR #146 OR #147 OR #148 OR #149 OR #150 OR #151 OR #152 OR #153 OR #154 OR #155 OR #156 OR #157 OR #158 OR #159 OR #160 OR #161 OR #162 OR #163 OR #164 OR #165 OR #166 OR #167 OR #168 OR #169 OR #170 OR #171 OR #172 OR #173 OR #174 OR #175 OR #176 OR #177 OR #178 OR #179 OR #180 OR #181 OR #182 OR #183 OR #184 OR #185) 396061

#187 #93 AND #118 AND #186 5795

#188 (randomized controlled trial):pt 498138

#189 (controlled clinical trial):pt 323841

#190 (randomized):ti,ab,kw 901465

#191 (placebo):ti,ab,kw 307102

#192 (randomly):ti,ab,kw 247614

#193 (trial):ti,ab,kw 825756

#194 (groups):ti,ab,kw 475957

#195 MeSH descriptor: [] explode all trees and with qualifier(s): [drug therapy - DT] 202047

#196 (#188 OR #189 OR #190 OR #191 OR #192 OR #193 OR #194 OR #195) 1349389

#197 #93 AND #118 AND #186 AND #196 with Publication Year from 2019 to 2020, in Trials 801

**CENTRAL (Cochrane)**

1. MeSH descriptor: [Osteoarthritis] explode all trees
2. (Osteoarthritis):ti,ab,kw
3. (Osteoarthrit*):ti,ab,kw
4. (Osteoarthros*):ti,ab,kw
5. 1 OR 2 OR 3 OR 4
6. (co-existing health problem*):ti,ab,kw
7. (coexisting illness*):ti,ab,kw
8. (co-existing illness*):ti,ab,kw
9. (coexisting patholog*):ti,ab,kw
10. (co-existing patholog*):ti,ab,kw
11. (comorbid condition*):ti,ab,kw
12. (co-morbid condition*):ti,ab,kw
13. (comorbid diagnos*):ti,ab,kw
14. (co-morbid diagnos*):ti,ab,kw
15. (comorbid disease*):ti,ab,kw
16. (co-morbid disease*):ti,ab,kw
17. (comorbid illness*):ti,ab,kw
18. (concurrent disease*):ti,ab,kw
19. (co-occurring condition*):ti,ab,kw
20. (cooccurring condition*):ti,ab,kw
21. ("multiple conditions"):ti,ab,kw
22. ("multiple disease"):ti,ab,kw
23. ("multiple diseases"):ti,ab,kw
24. MeSH descriptor: [Comorbidity] explode all trees
25. (Comorbidity):ti,ab,kw
26. ("comorbidities"):ti,ab,kw
27. (multimorbidity):ti,ab,kw
28. MeSH descriptor: [Multimorbidity] explode all trees
29. (multi-morbidity):ti,ab,kw
30. (multi-morbidities):ti,ab,kw
31. 6 OR 7 OR 8 OR 9 OR 10 OR 11 OR 12 OR 13 OR 14 OR 15 OR 16 OR 17 OR 18 OR 19 OR 20 OR 21 OR 22 OR 23 OR 24 OR 25 OR 26 OR 27 OR 28 OR 29 OR 30
32. MeSH descriptor: [Diabetes Mellitus] explode all trees
33. (diabetes mellitus):ti,ab,kw
34. MeSH descriptor: [Diabetes Mellitus, Type 2] explode all trees
35. (NIDDM):ti,ab,kw
36. (impaired glucose toleranc*):ti,ab,kw
37. MeSH descriptor: [Glucose Intolerance] explode all trees
38. MeSH descriptor: [Blood Glucose] explode all trees
39. 32 OR 33 OR 34 OR 35 OR 36 OR 37 OR 38
40. MeSH descriptor: [Depression] explode all trees
41. (depression):ti,ab,kw
42. MeSH descriptor: [Dysthymic Disorder] explode all trees
43. (Dysthymic Disorder):ti,ab,kw
44. (depress*):ti,ab,kw
45. (dysthymi*):ti,ab,kw
46. (affect disorder*):ti,ab,kw
47. (affective symptom*):ti,ab,kw
48. 40 OR 41 OR 42 OR 43 OR 44 OR 45 OR 46 OR 47
49. MeSH descriptor: [Hypertension] explode all trees
50. (Hypertension):ti,ab,kw
51. (Hypertens*):ti,ab,kw
52. (High Blood Pressure):ti,ab,kw
53. MeSH descriptor: [Blood Pressure] explode all trees
54. 49 OR 50 OR 51 OR 52 OR 53
55. MeSH descriptor: [Pulmonary Disease, Chronic Obstructive] explode all trees
56. MeSH descriptor: [Pulmonary Disease, Chronic Obstructive] explode all trees
57. (COPD):ti,ab,kw
58. MeSH descriptor: [Pulmonary Emphysema] explode all trees
59. (Pulmonary Emphysema):ti,ab,kw
60. MeSH descriptor: [Pulmonary Disease, Chronic Obstructive] explode all trees
61. (COAD):ti,ab,kw
62. MeSH descriptor: [Bronchitis, Chronic] explode all trees
63. (Chronic Bronchitis):ti,ab,kw
64. (Chronic Obstructive Lung Disease):ti,ab,kw
65. 55 OR 56 OR 57 OR 58 OR 59 OR 60 OR 61 OR 62 OR 63 OR 64
66. MeSH descriptor: [Myocardial Ischemia] explode all trees
67. (Myocardial Ischemia):ti,ab,kw
68. MeSH descriptor: [Coronary Artery Disease] explode all trees
69. (Coronary Artery Disease):ti,ab,kw
70. MeSH descriptor: [Coronary Disease] explode all trees
71. (Coronary Disease):ti,ab,kw
72. MeSH descriptor: [Myocardial Infarction] explode all trees
73. (myocardial infarction):ti,ab,kw
74. MeSH descriptor: [Angina Pectoris] explode all trees
75. (Angina Pectoris):ti,ab,kw
76. MeSH descriptor: [Heart Failure] explode all trees
77. (Heart Failure):ti,ab,kw
78. (HFNEF):ti,ab,kw
79. (HFPEF):ti,ab,kw
80. (HFREF):ti,ab,kw
81. ("HF PEF"):ti,ab,kw
82. MeSH descriptor: [Heart Diseases] explode all trees
83. (Heart Diseases):ti,ab,kw
84. MeSH descriptor: [Coronary Artery Bypass] explode all trees
85. (Coronary Artery Bypass):ti,ab,kw
86. 66 OR 67 OR 68 OR 69 OR 70 OR 71 OR 72 OR 73 OR 74 OR 75 OR 76 OR 77 OR 78 OR 79 OR 80 OR 81 OR 82 OR 83 OR 84 OR 85
87. 5 AND (31 OR 39 OR 48 OR 54 OR 65 OR 86)
88. 39 AND (31 OR 5 OR 48 OR 54 OR 65 OR 86)
89. 48 AND (31 OR 5 OR 39 OR 54 OR 65 OR 86)
90. 54 AND (31 OR 5 OR 39 OR 48 OR 65 OR 86)
91. 65 AND (31 OR 5 OR 39 OR 48 OR 54 OR 86)
92. 86 AND (31 OR 5 OR 39 OR 48 OR 54 OR 65)
93. (87 OR 88 OR 89 OR 90 OR 91 OR 92)
94. MeSH descriptor: [Health Behavior] explode all trees
95. MeSH descriptor: [Patient Education as Topic] explode all trees
96. MeSH descriptor: [Self Care] explode all trees
97. MeSH descriptor: [Self-Management] explode all trees
98. MeSH descriptor: [Disease Management] explode all trees
99. MeSH descriptor: [Health Promotion] explode all trees
100. MeSH descriptor: [Social Support] explode all trees
101. MeSH descriptor: [Behavior Therapy] explode all trees
102. MeSH descriptor: [Self Efficacy] explode all trees
103. MeSH descriptor: [Motivation] explode all trees
104. MeSH descriptor: [Primary Prevention] explode all trees
105. (behaviour* AND change):ti,ab,kw
106. (behaviour* AND technique):ti,ab,kw
107. (behaviour* AND intervention):ti,ab,kw
108. (behaviour* AND health):ti,ab,kw
109. (education* AND program):ti,ab,kw
110. (education* AND information):ti,ab,kw
111. (education* AND health):ti,ab,kw
112. (health promotion):ti,ab,kw
113. (coping):ti,ab,kw
114. (social support):ti,ab,kw
115. (self management):ti,ab,kw
116. (motivation):ti,ab,kw
117. (self-efficacy):ti,ab,kw
118. (94 OR 95 OR 96 OR 97 OR 98 OR 99 OR 100 OR 101 OR 102 OR 103 OR 104 OR 105 OR 106 OR 107 OR 108 OR 109 OR 110 OR 111 OR 112 OR 113 OR 114 OR 115 OR 116 OR 117)
119. 93 AND 118
120. MeSH descriptor: [Exercise] explode all trees
121. MeSH descriptor: [Sports] explode all trees
122. (Sports):ti,ab,kw
123. (Work-out):ti,ab,kw
124. (Workout):ti,ab,kw
125. (Working out):ti,ab,kw
126. (Aerobics):ti,ab,kw
127. MeSH descriptor: [Physical Fitness] explode all trees
128. (Fitness):ti,ab,kw
129. (Physical conditioning):ti,ab,kw
130. (Physically fit):ti,ab,kw
131. (Active lifestyle):ti,ab,kw
132. (Physical activity):ti,ab,kw
133. (Exercise):ti,ab,kw
134. MeSH descriptor: [Exercise Movement Techniques] explode all trees
135. (Exercise movement techniques):ti,ab,kw
136. MeSH descriptor: [Exercise Therapy] explode all trees
137. (Exercise therapy):ti,ab,kw
138. MeSH descriptor: [Locomotion] explode all trees
139. (Locomotion):ti,ab,kw
140. MeSH descriptor: [Physical Therapy Modalities] explode all trees
141. (Physical Therapy Modalities):ti,ab,kw
142. (Physiotherapy):ti,ab,kw
143. (Physical therapy):ti,ab,kw
144. MeSH descriptor: [Physical Education and Training] explode all trees
145. (Physical Education AND Training):ti,ab,kw
146. MeSH descriptor: [Motor Activity] explode all trees
147. (Motor Activity):ti,ab,kw
148. ("resistance training"):ti,ab,kw
149. (Strengthening):ti,ab,kw
150. MeSH descriptor: [Muscle Strength] explode all trees
151. (Muscle strength):ti,ab,kw
152. (Walking):ti,ab,kw
153. (Running):ti,ab,kw
154. (Jogging):ti,ab,kw
155. (Cycling):ti,ab,kw
156. (Bicycling):ti,ab,kw
157. (Swimming):ti,ab,kw
158. (Gymnastics):ti,ab,kw
159. (Yoga):ti,ab,kw
160. (Dancing):ti,ab,kw
161. MeSH descriptor: [Dancing] explode all trees
162. (Pilates):ti,ab,kw
163. MeSH descriptor: [Body Mass Index] explode all trees
164. (Body Mass Index):ti,ab,kw
165. MeSH descriptor: [Body Weight] explode all trees
166. (Body weight):ti,ab,kw
167. (Weight loss):ti,ab,kw
168. (Overweight):ti,ab,kw
169. (Obesity):ti,ab,kw
170. MeSH descriptor: [Health Promotion] explode all trees
171. (Health promotion):ti,ab,kw
172. (Adiposity):ti,ab,kw
173. MeSH descriptor: [Adiposity] explode all trees
174. (Excess body fat):ti,ab,kw
175. MeSH descriptor: [Activities of Daily Living] explode all trees
176. (Activities of daily living):ti,ab,kw
177. (ADL):ti,ab,kw
178. (functional ability):ti,ab,kw
179. (physical limitations):ti,ab,kw
180. (physical function):ti,ab,kw
181. (functional assessment):ti,ab,kw
182. MeSH descriptor: [Health Status] explode all trees
183. (Health status):ti,ab,kw
184. (Quality of life):ti,ab,kw
185. (HRQOL):ti,ab,kw
186. (120 OR 121 OR 122 OR 123 OR 124 OR 125 OR 126 OR 127 OR 128 OR 129 OR 130 OR 131 OR 132 OR 133 OR 134 OR 135 OR 136 OR 137 OR 138 OR 139 OR 140 OR 141 OR 142 OR 143 OR 144 OR 145 OR 146 OR 147 OR 148 OR 149 OR 150 OR 151 OR 152 OR 153 OR 154 OR 155 OR 156 OR 157 OR 158 OR 159 OR 160 OR 161 OR 162 OR 163 OR 164 OR 165 OR 166 OR 167 OR 168 OR 169 OR 170 OR 171 OR 172 OR 173 OR 174 OR 175 OR 176 OR 177 OR 178 OR 179 OR 180 OR 181 OR 182 OR 183 OR 184 OR 185)
187. 93 AND 118 AND 186
188. (randomized controlled trial):pt
189. (controlled clinical trial):pt
190. (randomized):ti,ab,kw
191. (placebo):ti,ab,kw
192. (randomly):ti,ab,kw
193. (trial):ti,ab,kw
194. (groups):ti,ab,kw
195. MeSH descriptor: [] explode all trees and with qualifier(s): [drug therapy - DT]
196. (188 OR 189 OR 190 OR 191 OR 192 OR 193 OR 194 OR 195)
197. 93 AND 118 AND 186 AND 196 with Publication Year from 2019 to 2020, in Trials

## Additional File 2 PRISMA 2020 flow chart.

**Identification of studies via other methods**

**Identification of studies via databases and registers**

Records identified from:

Websites (n = 0)

Organisations (n = 0)

Citation searching (n = 72)

etc.

Records removed *before screening*:

Duplicate records removed
(n = 137)

Records marked as ineligible by automation tools (n = 0)

Records removed for other reasons (n = 0)

Records identified from*:

Databases (n = 1226)

Registers (n = 0)

**Identification**

Records screened

(n = 1089)

Records excluded**

(n = 1072)

Reports not retrieved

(n = 0)

Reports sought for retrieval

(n = 72)

Reports sought for retrieval

(n = 17)

Reports not retrieved

(n = 0)

**Screening**

Reports excluded:

Wrong patient population
(n = 10)

Wrong intervention (n = 2)

Wrong language (n = 1)

Wrong study design (n = 1)

Duplicate (n = 1)

Reports assessed for eligibility

(n = 72)

Reports excluded:

Wrong patient population
(n = 60)

Reports assessed for eligibility

(n = 17)

Studies included in review

(n = 14)

Reports of included studies

(n = 0)

**Included**

*Consider, if feasible to do so, reporting the number of records identified from each database or register searched (rather than the total number across all databases/registers).

**If automation tools were used, indicate how many records were excluded by a human and how many were excluded by automation tools.

*From:*  Page MJ, McKenzie JE, Bossuyt PM, Boutron I, Hoffmann TC, Mulrow CD, et al. The PRISMA 2020 statement: an updated guideline for reporting systematic reviews. BMJ 2021;372:n71. doi: 10.1136/bmj.n71. For more information, visit: <http://www.prisma-statement.org/>

**Figure 1**. Flow chart of the included studies.

## Additional File 3. Table 1 (Behavior Change Techniques used in the individual studies)

|  | Behavior Change Techniques | | | | | | | | | | | | | | | | | | | | | | | | | | | | | | | | | | |
| --- | --- | --- | --- | --- | --- | --- | --- | --- | --- | --- | --- | --- | --- | --- | --- | --- | --- | --- | --- | --- | --- | --- | --- | --- | --- | --- | --- | --- | --- | --- | --- | --- | --- | --- | --- |
| Author and year | **1.1** | **1.2** | **1.3** | **1.4** | **1.5** | **1.7** | **1.8** | **2.1** | **2.3** | **2.4** | **2.5** | **2.6** | **2.7** | **3.1** | **3.2** | **3.3** | **4.1** | **4.4** | **5.1** | **5.3** | **5.6** | **6.1** | **6.2** | **7.1** | **8.1** | **8.6** | **8.7** | **9.1** | **11.1** | **11.2** | **12.5** | **12.6** | **13.2** | **13.4** | **Tot** |
| Koukouvou 2004 |  |  |  | 1 |  |  |  |  |  | 1 |  |  |  |  |  |  | 1 |  |  |  |  | 1 |  |  | 1 |  | 1 |  |  |  |  |  |  |  | 6 |
| Kulcu 2007 |  |  |  | 1 |  |  |  |  |  |  |  |  |  | 1 |  |  | 1 |  |  |  |  | 1 |  |  | 1 |  | 1 | 1 |  |  |  |  |  |  | 7 |
| Gary 2010a | 1 |  |  | 1 |  |  |  | 1 | 1 | 1 |  | 1 |  | 1 |  |  | 1 |  | 1 |  |  | 1 |  |  |  |  | 1 |  |  |  | 1 |  |  |  | 12 |
| Gary 2010b | 1 |  | 1 | 1 |  |  |  | 1 | 1 | 1 |  | 1 |  | 1 |  |  | 1 |  | 1 |  |  | 1 |  |  |  |  | 1 |  |  |  | 1 |  | 1 |  | 14 |
| Katon 2010 | 1 | 1 | 1 |  |  |  |  |  |  | 1 | 1 |  |  | 1 | 1 |  |  |  |  |  |  | 1 |  |  |  |  |  |  | 1 |  | 1 |  |  |  | 1 |
| Piette 2011 |  |  | 1 | 1 |  |  |  |  |  | 1 | 1 |  |  | 1 | 1 |  | 1 |  |  |  | 1 |  |  |  |  |  |  |  |  |  | 1 |  |  |  | 9 |
| Åsa 2012 |  |  |  | 1 |  |  |  |  |  |  |  | 1 |  |  |  |  | 1 |  |  |  |  | 1 |  |  | 1 |  |  |  |  |  |  |  |  |  | 5 |
| Lynch 2014 |  | 1 | 1 |  |  |  |  |  | 1 | 1 |  |  |  | 1 |  | 1 | 1 |  |  |  |  | 1 | 1 |  |  |  |  |  |  |  | 1 |  | 1 |  | 11 |
| Pibernik-Okanović 2015 |  |  |  |  |  |  |  |  |  |  | 1 | 1 |  |  |  |  | 1 |  |  |  |  |  |  |  | 1 |  |  |  |  |  |  |  |  |  | 4 |
| Pibernik-Okanović 2015 |  |  |  |  |  |  |  |  |  |  | 1 | 1 |  |  |  |  | 1 |  |  |  |  |  |  |  | 1 |  |  |  |  |  |  |  |  |  | 4 |
| Keihani 2015 |  |  |  | 1 |  |  |  |  |  | 1 |  |  |  | 1 |  |  | 1 |  |  |  |  | 1 |  |  | 1 |  | 1 |  |  |  |  |  |  |  | 7 |
| Dunbar 2015 |  | 1 | 1 |  |  |  |  |  |  | 1 | 1 |  |  | 1 | 1 | 1 | 1 |  | 1 | 1 |  |  |  | 1 |  |  |  |  |  |  |  |  |  |  | 11 |
| Freedland 2015 |  | 1 | 1 |  |  | 1 |  |  |  |  | 1 |  |  | 1 |  |  |  |  |  |  |  |  |  |  |  |  |  |  |  |  |  |  |  |  | 5 |
| Schneider 2016 |  | 1 |  |  |  |  | 1 |  | 1 | 1 |  | 1 |  | 1 |  |  | 1 | 1 |  |  |  | 1 |  |  | 1 | 1 | 1 |  |  |  | 1 | 1 |  | 1 | 15 |
| Huang 2016 |  | 1 |  |  |  |  |  |  |  |  |  |  | 1 | 1 |  | 1 | 1 |  |  |  |  | 1 | 1 |  | 1 |  |  |  |  | 1 |  |  |  |  | 9 |
| de Groot 2019a | 1 |  |  | 1 | 1 |  |  |  | 1 |  | 1 |  |  |  | 1 |  | 1 |  |  |  |  | 1 |  |  | 1 |  | 1 |  |  |  |  |  |  |  | 1 |
| de Groot 2019b | 1 |  |  | 1 | 1 |  |  |  | 1 |  | 1 |  |  | 1 | 1 |  | 1 |  |  |  |  | 1 |  |  | 1 |  | 1 |  |  |  |  |  | 1 |  | 12 |

1.1. Goal setting (behavior), 1.2. Problem solving, 1.3. Goal setting (outcome), 1.4. Action planning, 1.5. Review behavior goal(s), 1.7. Review outcome goal(s) 1.8. Behavioral contract, 2.1. Monitoring of behavior by others without feedback, 2.3. Self-monitoring of behaviour 2.4. Self-monitoring of outcome(s) of behaviour, 2.5. Monitoring of outcome(s) of behavior without feedback, 2.6. Biofeedback 2.7. Feedback on outcome(s) of behavior, 3.1. Social support (unspecified), 3.2. Social support (practical), 3.3. Social support (emotional), 4.1. Instruction on how to perform the behavior, 4.4. Behavioral experiments, 5.1. Information about health consequences, 5.3. Information about social and environmental consequences, 5.6. Information about emotional consequences, 6.1. Demonstration of the behavior, 6.2. Social comparison, 7.1. Prompts/cues, 8.1. Behavioral practice/rehearsal, 8.6. Generalisation of target behavior, 8.7. Graded tasks, 9.1. Credible source, 11.1. Pharmacological support, 11.2. Reduce negative emotions, 12.5. Adding objects to the environment, 12.6. Body changes, 13.2. Framing/reframing, 13.4. Valued self-identify.

## Additional File 4. BCT effectiveness ratio summary

|  | **Physical activity** | | **Weight loss** | |
| --- | --- | --- | --- | --- |
|  | **End-treatment** | **Effectiveness Ratio (%)** | **End-treatment** | **Effectiveness Ratio (%)** |
| **Problem solving 1.2** | 1 (=) 3 (↑) | 75 | 2 (=) 1 (↑) | 33 |
| **Goal setting (outcome)**  **1.3** | 1 (=) 4 (↑) | 80 | NA | NA |
| **Action planning 1.4** | 3 (↑) | 100 | 3 (↓) | 100 |
| **Self-monitoring of behavior 2.3** | NA | NA | 1 (=)  3 (↓) | 75 |
| **Self-monitoring of outcome(s) of behavior 2.4** | 1 (=) 5 (↑) | 84 | 1 (=) 2 (↓) | 66 |
| **Monitoring outcome(s) of behavior by others without feedback 2.5** | 1 (=) 3 (↑) | 75 | 2 (↓) 1 (↑) | 66 |
| **Social support (unspecified) 3.1** | 2 (=) 5 (↑) | 72 | 2 (↓)  2 (=) 1 (↑) | 40 |
| **Social support (practical)**  **3.2** | 3 (↑) | 100 | 3 (↓) | 100 |
| **Instruction on how to perform a behavior 4.1** | 1 (=) 5 (↑) | 84 | 2 (=) 4 (↓) | 66 |
| **Demonstration of the behavior 6.1** | 1 (=) 4 (↑) | 75 | 2 (=) 4 (↓) | 67 |
| **Behavioral practice/ rehearsal 8.1** | 1 (=) 2 (↑) | 66 | 1 (=) 3 (↓) | 60 |
| **Graded tasks 8.7** | 1 (=) 2 (↑) | 66 | 1 (=) 3 (↓) | 75 |
| **Adding objects to the environment**  **12.5** | 1 (=) 3 (↑) | 75 | NA | NA |

## Additional File 4. Weight change meta-analysis at long term follow-up


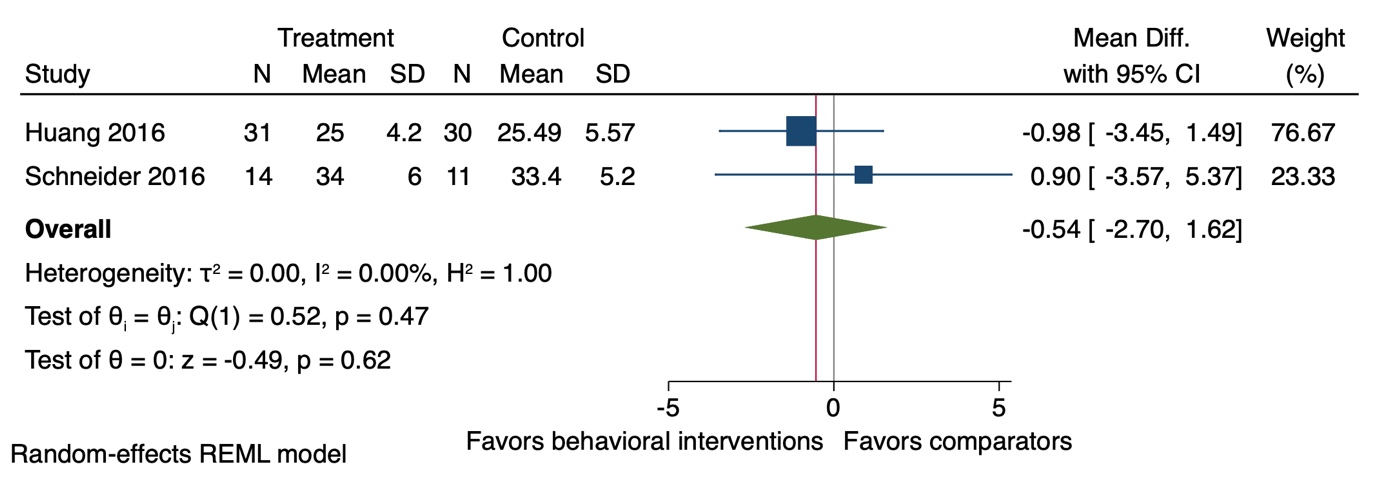


**Figure AF 4**. Forest plot for the effect of behavioural interventions compared to a usual care comparator group on weight loss at long-term follow-up. 95 % CI = 95 % Confidence Interval.

## Additional File 5. Health-related quality of life meta-analysis at long term follow-up


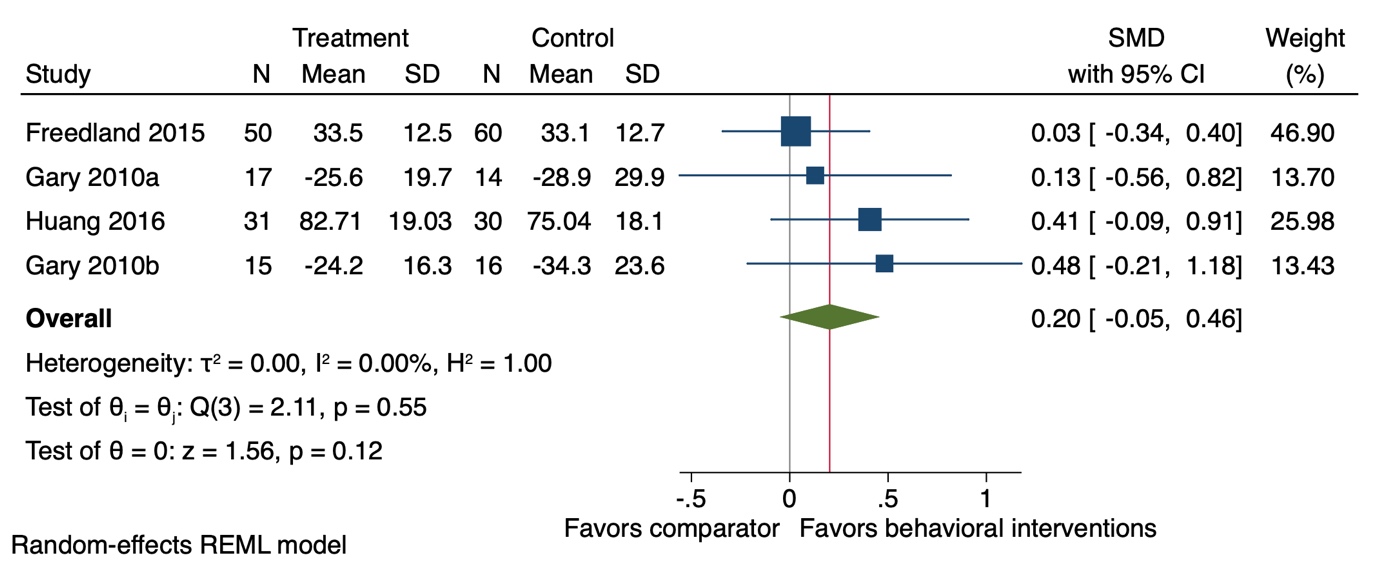


**Figure AF 5**. Forest plot for the effect of behavioural interventions compared to a usual care comparator group on health-related quality of life at long-term follow-up. SMD = Standardised Mean Difference; 95 % CI = 95 % Confidence Interval. a,b=two separate study comparisons from the same study.

## Additional File 6. Meta-regression participants, trials, interventions and BCT characteristics

|  | Physical function | | | | | | Depression | | | | |  |
| --- | --- | --- | --- | --- | --- | --- | --- | --- | --- | --- | --- | --- |
|  | **Number of comparisons** | **Effect size (SMD)** | **95%CI low** | **95%CI high** | **tau2** | **Number of comparisons** | | **Effect size (SMD)** | **95%CI low** | **95%CI high** | **tau2** |  |
|  | 9 | 0.42 | 0.12 | 0.73 | 0.13 | 13 | | -0.70 | -0.98 | -0.42 | 0.17 |  |
|  | **Number of comparisons** | **Coef.** | **95%CI low** | **95%CI high** | **tau2** | **Number of comparisons** | | **Coef.** | **95%CI low** | **95%CI high** | **tau2** |  |
| PARTICIPANT CHARACTERISTICS | | | | | | | | | | | | |
| Age | **9 (slope)** | **0.08** | **0.02** | **0.13** | **0.05** | 13 (slope) | | 0.03 | -0.04 | 0.10 | 0.19 |  |
| Female proportion | **9 (slope)** | **-0.02** | **-0.04** | **-0.01** | **0.09** | 13 (slope) | | 0.01 | -0.01 | 0.02 | 0.17 |  |
| BMI | 5 (slope) | -0.07 | -0.16 | 0.02 | 0.16 | **8 (slope)** | | **0.09** | **0.04** | **0.15** | **0.08** |  |
| Depression severity | 7 (slope) | 0.02 | -0.01 | 0.05 | 0.08 | 12 (slope) | | 0.01 | -0.03 | 0.03 | 0.22 |  |
| INTERVENTION CHARACTERISTICS | | | | | | | | | | | | |
| Frequency (sessions per week) | 9 (slope) | 0.23 | -0.07 | 0.52 | 0.10 | 13 (slope) | | -0.40 | -0.32 | 0.24 | 0.19 |  |
| Mode of delivery  (Individual vs Group) | 9 (ref Group)) | -0.13 | -0.85 | 0.60 | 0.16 | 13 (slope) | | -0.41 | -0.95 | 0.13 | 0.14 |  |
| Intervention length (in weeks) | 9 (slope) | -0.03 | -0.08 | 0.02 | 0.13 | 13 (slope) | | 0.01 | -0.02 | 0.03 | 0.19 |  |
| BEHAVIOR CHANGE TECHNIQUES | | | | | | | | | | | | |
| 1.1 Goal setting (behavior) | Ref (no BCT 1.1) (k=5) | -0.30 | -0.96 | 0.36 | 0.15 | Ref (no BCT 1.1) (k=8) | | 0.30 | -0.29 | 0.89 | 0.18 |  |
| 1.2 Problem solving (behavior) | Ref (no BCT 1.2) (k=7) | -0.23 | -0.96 | 0.51 | 0.16 | Ref (no BCT 1.2) (k=10) | | -0.39 | -0.66 | 0.59 | 0.20 |  |
| 1.3 Goal setting (outcome) | Ref (no BCT 1.3) (k=5) | -0.12 | -0.77 | 0.54 | 0.16 | Ref (no BCT 1.3) (k=5) | | 0.43 | -0.42 | 0.97 | 0.12 |  |
| 1.4 Action planning | Ref (no BCT 1.4) (k=5) | -0.30 | -0.97 | 0.36 | 0.15 | Ref (no BCT 1.4) (k=4) | | 0.04 | -0-59 | 0.66 | 0.21 |  |
| 2.3 Self-monitoring of behavior | Ref (no BCT 2.3) (k=5) | -0.30 | -0.97 | 0.36 | 0.15 | Ref (no BCT 2.3) (k=8) | | 0.37 | -0.23 | 0.97 | 0.18 |  |
| 2.4 Self-monitoring of outcome(s) of behavior | Ref (no BCT 2.4) (k=4) | 0.40 | -0.15 | 0.95 | 0.09 | Ref (no BCT 2.4) (k=7) | | 0.33 | -0.23 | -0.89 | 0.17 |  |
| 2.5 Monitoring of outcomes of behavior without feedback | **Ref (no BCT 2.5) (k=4)** | **-0.69** | **-1.16** | **-0.22** | **0.30** | Ref (no BCT 2.5) (k=9) | | 0.30 | -0.24 | 0.85 | 0.15 |  |
| 3.1 Social support (unspecified) | Ref (no BCT 3.1) (k=2) | -0.21 | -1.10 | 0.66 | 0.16 | Ref (no BCT 3.1) (k=3) | | 0.14 | -0.61 | 0.89 | 0.19 |  |
| 3.2 Social support (practical) | Ref (no BCT 3.2) (k=5) | -0.361 | -1.00 | 0.29 | 0.15 | Ref (no BCT 3.2) (k=9) | | 0.19 | -0.41 | 0.79 | 0.19 |  |
| 6.1 Demonstration of the behavior | Ref (no BCT 6.1) (k=3) | 0.28 | -0.35 | 0.91 | 0.14 | Ref (no BCT 6.1) (k=2) | | -0.36 | -1.04 | 0.32 | 0.17 |  |
| 8.1 Behavioral practice/rehearsal | Ref (no BCT 8.1) (k=5) | 0.22 | -0.43 | 0.88 | 0.15 | Ref (no BCT 8.1) (k=4) | | -0.57 | -1.00 | -0.15 | 0.07 |  |
| 8.7 Graded tasks | Ref (no BCT 8.7) (k=4) | 0.23 | -0.64 | 0.69 | 0.17 | Ref (no BCT 8.7) (k=4) | | 0.01 | -0.59 | 0.60 | 0.20 |  |
| 12.5 Adding objects to the environment | Ref (no BCT 12.5) (k=6) | -0.04 | -0.75 | 0.67 | 0.17 | Ref (no BCT 12.5) (k=8) | | 0.60 | 0.12 | 1.09 | 0.10 |  |
| Total number of BCT for Goal setting and planning | **9 (slope)** | **-0.45** | **-0.73** | **-0.18** | **0.02** | **13 (slope)** | | **0.31** | **0.04** | **0.58** | **0.10** |  |
| Total number of BCT for Feedback and monitoring | 9 (slope) | -0.04 | -0.37 | 0.29 | 0.17 | **13 (slope)** | | **0.25** | **0.02** | **0.48** | **0.11** |  |
| Total number of BCT for Social support | 9 (slope) | -0.16 | -0.58 | 0.26 | 0.17 | 13 (slope) | | -0.09 | -0.55 | 0.37 | 0.19 |  |
| Total number of BCT for Natural consequences | 9 (slope) | 0.07 | -0.40 | 0.55 | 0.17 | Ref (no BCT for Natural consequences) (k=2) | | 0.48 | -0.16 | 1.13 | 0.16 |  |
| Total number of BCTs | 9 (slope) | -0.02 | -0.14 | 0.10 | .017 | 13 (slope) | | 0.05 | -0.04 | 0.15 | 0.18 |  |

Data for continuous covariates tested in meta-regression analyses were reported in at least 5 studies included in meta-analysis. Data for categorical covariates were tested in meta-regression analyses when there were at least two studies as a reference category.

Ref=reference category.

## Additional File 7. Sub-group analysis on physical function


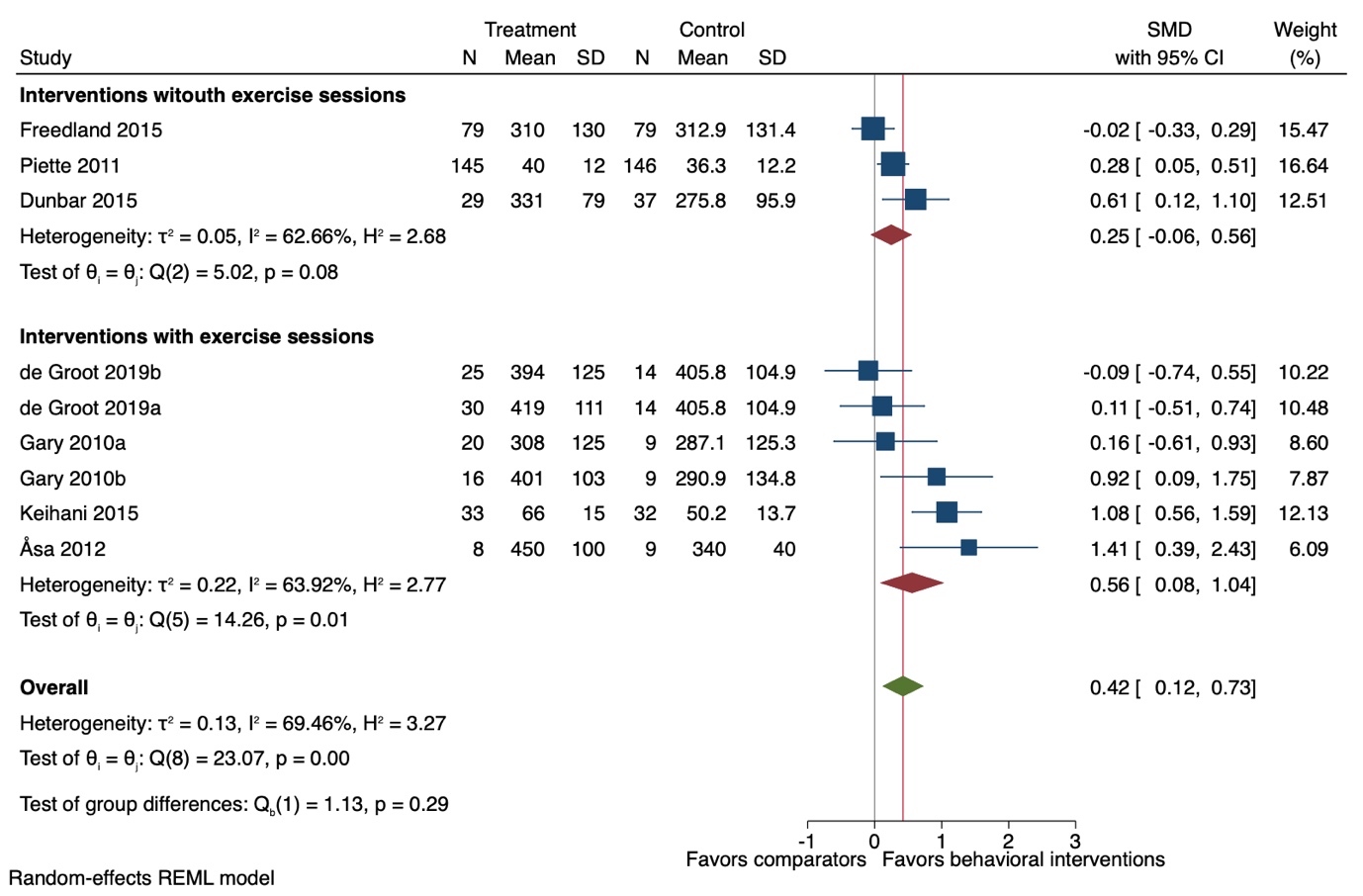


**Figure AF 3.** Forest plot for the effect of behavioural interventions compared to a usual care comparator group on physical function stratified by components of the interventions. SMD = Standardised Mean Difference; 95 % CI = 95 % Confidence Interval. ^a,b^=two separate study comparisons from the same study.

## Additional File 8. Risk of Bias summary


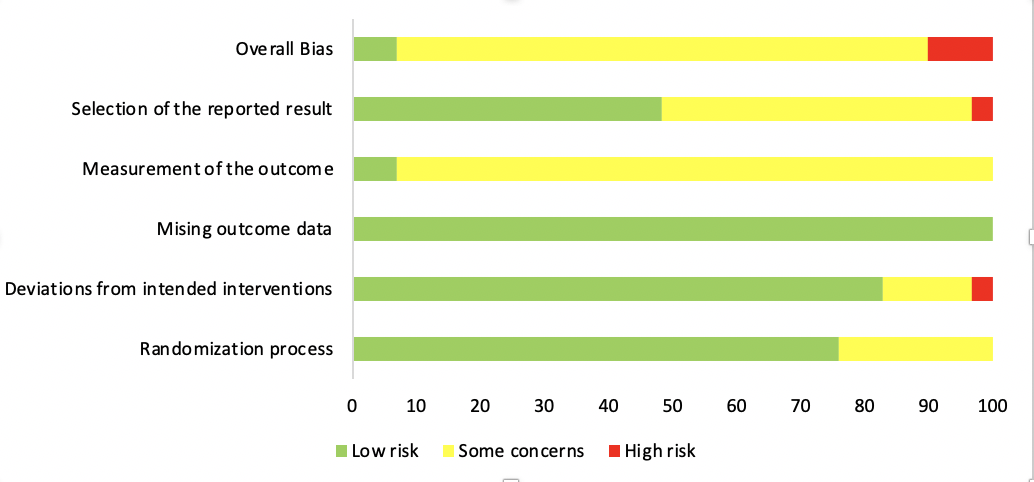


**Figure AF 4.** 'Risk of bias’ summary of six individual ‘Risk of bias’ items for each included study.

## Additional File 9. Small study bias.

a)
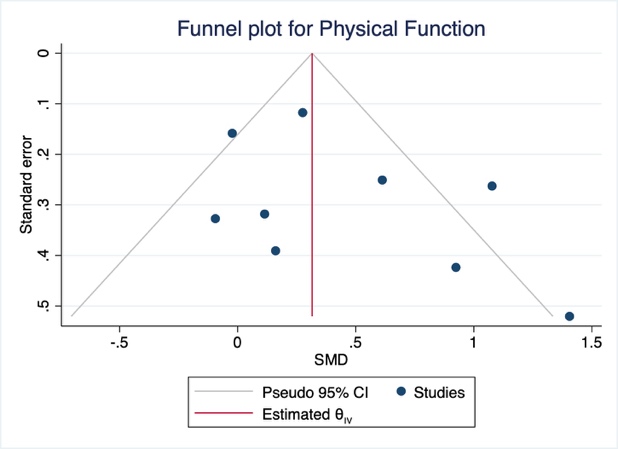


b)
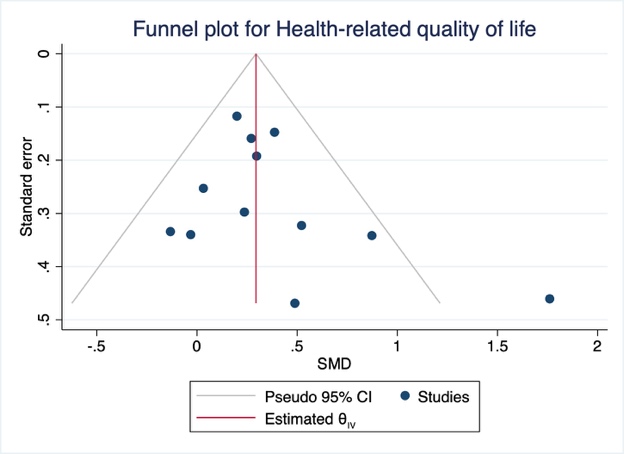


c)
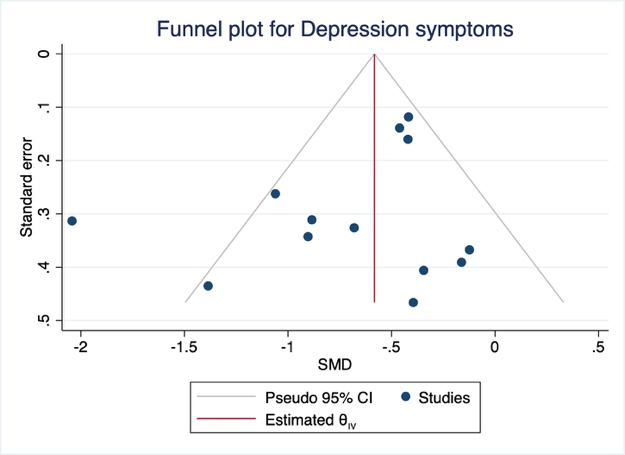


**Supplementary figure 9**: Funnel plots investigating small study bias for the outcomes Health-related quality of life (a), physical function (b) and depression symptoms (c)

## Additional File 10. STATA commands to reproduce the analyses

//do file for the systematic review "Effect of behavioural interventions in people with multimorbidity: systematic review and meta-analysis" Bricca et al. 2021

*STATA output to reproduce meta-analyses and meta-regression analyses

//import dataset first//

//Meta-analyses for the outcomes: physical activity, weight change, physical function and health-related quality of life at the end-treatment timepoint

keep if Timepoint == 1

//Physical activity obsevtively measured (Figure 1)

meta esize n_oPA_IG mean_int_oPA SD_int_oPA n_oPA_CG mean_con_oPA SD_con_oPA, esize(hedges) eslabel(SMD) studylabel(Author)

meta forestplot, random esrefline nullrefline(favorsr("Favors behavioral interventions") favorsl("Favors comparators")) columnopts(_mean1 _sd1 _mean2 _sd2, format(%9.1f)) sort(_meta_es)

//Weight change (Figure 3)

meta esize n_WL_IG mean_int_WL SD_int_WL n_WLA_CG mean_con_WL SD_con_WL, esize(mdiff) studylabel(Author)

meta forestplot, random esrefline nullrefline(favorsr("Favors comparators") favorsl("Favors behavioral interventions")) columnopts(_mean1 _sd1 _mean2 _sd2, format(%9.1f)) sort(_meta_es)

//Health-related quality of life (Figure 5)

meta esize n_HRQL_IG mean_int_HRQL SD_int_HRQL n_HRQL_CG mean_con_HRQL SD_con_HRQL, esize(hedges) eslabel(SMD) studylabel(Author)

meta forestplot, random esrefline nullrefline(favorsr("Favors behavioral interventions") favorsl("Favors comparators")) columnopts(_mean1 _sd1 _mean2 _sd2, format(%9.1f)) sort(_meta_es)

//Physical function (Figure 6)

meta esize n_PF_IG mean_int_PF SD_int_PF n_PF_CG mean_con_PF SD_con_PF, esize(hedges) eslabel(SMD) studylabel(Author)

meta forestplot, random esrefline nullrefline(favorsr("Favors behavioral interventions") favorsl("Favors comparators")) columnopts(_mean1 _sd1 _mean2 _sd2, format(%9.1f)) sort(_meta_es)

#################################################################################################################

//Additional meta-analysis on Depression at the end-treatment timepoint (Figure 7)

meta esize n_DEP_IG mean_int_DEP SD_int_DEP n_DEP_CG mean_con_DEP SD_con_DEP, esize(hedges) eslabel(SMD) studylabel(Author)

meta forestplot, random esrefline nullrefline(favorsr("Favors comparator") favorsl("Favors behavioral interventions")) columnopts(_mean1 _sd1 _mean2 _sd2, format(%9.1f)) sort(_meta_es, descending)

//Sentivity meta-analysis with physical activity and physical function together (Figure 8)

meta esize n_IG mean_IG_PAPF SD_IG_PAPF n_CG mean_CG_PAPF SD_CG_PAPF, esize(hedges) eslabel(SMD) studylabel(Author)

meta forestplot, random esrefline nullrefline(favorsr("Favors behavioral interventions") favorsl("Favors comparators")) columnopts(_mean1 _sd1 _mean2 _sd2, format(%9.1f)) sort(_meta_es, descending)

//Sentivity meta-analysis including the mental component scale data instead of the physical component score data for the studies using the SF-12 (Figure 9)

meta esize s_n_HRQL_IG s_mean_int_HRQL s_SD_int_HRQL s_n_HRQL_CG s_mean_con_HRQL s_SD_con_HRQL, esize(hedges) eslabel(SMD) studylabel(Author)

meta forestplot, random esrefline nullrefline(favorsr("Favors behavioral interventions") favorsl("Favors comparator")) columnopts(_mean1 _sd1 _mean2 _sd2, format(%9.1f)) sort(_meta_es, descending)

//Sub-group meta-analysis on physical function structures exercise vs. non-structured exercise interventions (Additional File 7)

meta esize n_PF_IG mean_int_PF SD_int_PF n_PF_CG mean_con_PF SD_con_PF, esize(hedges) eslabel(SMD) studylabel(Author)

meta forestplot, random esrefline nullrefline(favorsr("Favors behavioral interventions") favorsl("Favors comparators")) columnopts(_mean1 _sd1 _mean2 _sd2, format(%9.1f)) sort(_meta_es) subgroup(Exercise_vs_pa) nooverall

#################################################################################################################

//Meta-analyses for the outcomes: weight change and health-related quality of life at the follow-up closest to 12 months after randomisation

//import dataset first//

keep if Timepoint == 2

//Weight change (Additional figure 4)

meta esize n_WL_IG mean_int_WL SD_int_WL n_WLA_CG mean_con_WL SD_con_WL, esize(mdiff) studylabel(Author)

meta forestplot, random esrefline nullrefline(favorsr("Favors comparators") favorsl("Favors behavioral interventions")) columnopts(_mean1 _sd1 _mean2 _sd2, format(%9.1f)) sort(_meta_es)

//Health-related quality of life (Additional figure 5)

meta esize n_HRQL_IG mean_int_HRQL SD_int_HRQL n_HRQL_CG mean_con_HRQL SD_con_HRQL, esize(hedges) eslabel(SMD) studylabel(Author)

meta forestplot, random esrefline nullrefline(favorsr("Favors behavioral interventions") favorsl("Favors comparators")) columnopts(_mean1 _sd1 _mean2 _sd2, format(%9.1f)) sort(_meta_es)

#################################################################################################################

//Meta-regression analyses for the outcomes physical function and depression (Additional file 6)

//import dataset first//

keep if Timepoint == 1

//estimate effect sizes for physical function or depression and then run meta-regression analyses

//physical function

meta esize n_PF_IG mean_int_PF SD_int_PF n_PF_CG mean_con_PF SD_con_PF, esize(hedges) eslabel(SMD) studylabel(Author)

meta regress Age

meta regress _female

meta regress BMI

meta regress depression_severity_0_100

meta regress Freq_sessions_tw

meta regress MoD

meta regress Weeks_PR

meta regress BCT_01_01

meta regress BCT_01_02

meta regress BCT_01_03

meta regress BCT_01_04

meta regress BCT_02_05

meta regress BCT_03_01

meta regress BCT_03_02

meta regress BCT_06_01

meta regress BCT_08_01

meta regress BCT_08_07

meta regress BCT_12_05

meta regress BCT_01_tot

meta regress BCT_02_tot

meta regress BCT_03_tot

meta regress BCT_05_tot

meta regress BCT_TOTAL

//depression

meta esize n_DEP_IG mean_int_DEP SD_int_DEP n_DEP_CG mean_con_DEP SD_con_DEP, esize(hedges) eslabel(SMD) studylabel(Author)

meta regress Age

meta regress _female

meta regress BMI

meta regress depression_severity_0_100

meta regress Freq_sessions_tw

meta regress MoD

meta regress Weeks_PR

meta regress BCT_01_01

meta regress BCT_01_02

meta regress BCT_01_03

meta regress BCT_01_04

meta regress BCT_02_05

meta regress BCT_03_01

meta regress BCT_03_02

meta regress BCT_06_01

meta regress BCT_08_01

meta regress BCT_08_07

meta regress BCT_12_05

meta regress BCT_01_tot

meta regress BCT_02_tot

meta regress BCT_03_tot

meta regress BCT_05_tot

meta regress BCT_TOTAL

#################################################################################################################

//Publication bias (Additional file 9)

//Estimate effect sizes for physical function, health-related quality of life and depression and then run meta funnelplot

//physical function

meta esize n_PF_IG mean_int_PF SD_int_PF n_PF_CG mean_con_PF SD_con_PF, esize(hedges) eslabel(SMD) studylabel(Author)

meta funnelplot

//hrqol

meta esize n_HRQL_IG mean_int_HRQL SD_int_HRQL n_HRQL_CG mean_con_HRQL SD_con_HRQL, esize(hedges) eslabel(SMD) studylabel(Author)

meta funnelplot

//depression

meta esize n_DEP_IG mean_int_DEP SD_int_DEP n_DEP_CG mean_con_DEP SD_con_DEP, esize(hedges) eslabel(SMD) studylabel(Author)

meta funnelplot
